# Covalent Dynamic DNA Networks to Translate Multiple Inputs into Programmable Outputs

**DOI:** 10.1021/jacs.4c13854

**Published:** 2025-02-05

**Authors:** Simone Brannetti, Serena Gentile, Erica Del Grosso, Sijbren Otto, Francesco Ricci

**Affiliations:** † Department of Chemical Sciences and Technologies, University of Rome, Tor Vergata, Via della Ricerca Scientifica, Rome 00133, Italy; ‡ Centre for Systems Chemistry, Stratingh Institute, University of Groningen, Groningen 9747 AG, Netherlands

## Abstract

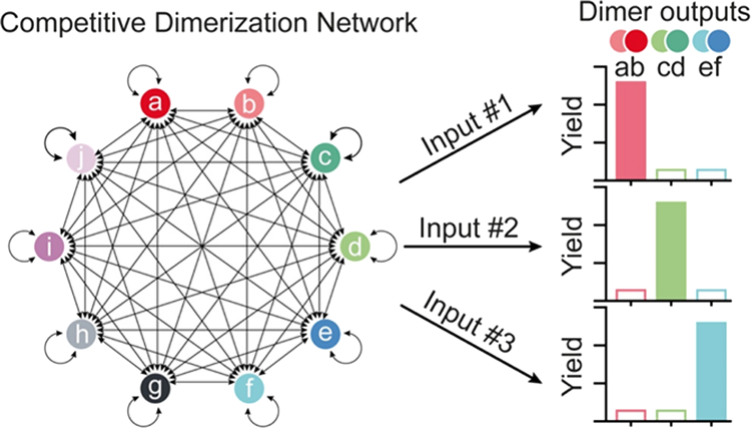

Inspired by naturally occurring protein dimerization networks, in which a set of proteins interact with each other to achieve highly complex input-output behaviors, we demonstrate here a fully synthetic DNA-based dimerization network that enables highly programmable input-output computations. Our DNA-based dimerization network consists of DNA oligonucleotide monomers modified with reactive moieties that can covalently bond with each other to form dimer outputs in an all-to-all or many-to-many fashion. By designing DNA-based input strands that can specifically sequester DNA monomers, we can control the size of the reaction network and thus fine-tune the yield of each DNA dimer output in a predictable manner. Thanks to the programmability and specificity of DNA–DNA interactions, we show that this approach can be used to control the yield of different dimer outputs using different inputs. The approach is also versatile and we demonstrate dimerization networks based on two distinct covalent reactions: thiol–disulfide and strain-promoted azide–alkyne cycloaddition (SPAAC) reactions. Finally, we show here that the DNA-based dimerization network can be used to control the yield of a functional dimer output, ultimately controlling the assembly and disassembly of DNA nanostructures. The covalent dynamic DNA networks shown here provide a way to convert multiple inputs into programmable outputs that can control a broader range of functions, including ones that mimic those of living cells.

## Introduction

The living cell is an impressive and inspiring example of how highly developed functions can emerge from a system of reacting and interacting molecules. While the inner workings of a cell are still being unraveled, there is growing interest in the development and construction of systems that perform some of the many functions of life from scratch using the same basic ingredients: the formation of covalent bonds and noncovalent interactions. These efforts have shaped the field of systems chemistry^1−3^ and have led to synthetic systems that can move,^4−6^ replicate,^7−9^ evolve^10,11^ and metabolize.^12,13^ Further development of this field requires systems that are capable of processing information and enabling communication between different components, which is necessary for their proper integration into higher-level systems.

The topic of how molecular networks that combine chemical reactions with noncovalent interactions can process information at the molecular level has received comparatively little attention. Examples include work on how molecular recognition events propagate through dynamic covalent reaction networks or combinatorial libraries^14−16^ in which simple monomer units oligomerize and reversibly exchange monomers.^17−20^ While these cases demonstrate the potential of dynamic molecular networks to respond to specific molecular inputs, they are somewhat limited in terms of programmability and predictability.^21^ The advancement of these aspects places ever-increasing demands on the specificity and tunability of molecular recognition events.

More recently, the exquisite predictability and sequence-specificity of DNA/DNA hybridization has enabled the construction of DNA-based reaction networks,^22^ nanostructures^23,24^ and circuits^25,26^ that can process different inputs to provide predictable and programmable outputs in a modular fashion.^27^ In these systems, multiple DNA-based reactions (e.g., strand displacement) are used to create logic gates^28,29^ and neural networks^30,31^ that can process inputs and deliver outputs through complex signaling pathways. Compartmentalization of these systems can also lead to a higher level of computation capabilities.^32−34^ DNA-based constitutional dynamic networks (CDNs) that enable adaptive behavior, increased dimensionality, and communication between different catalytic networks by mimicking natural dynamic signaling processes have also recently been demonstrated.^35−40^ The DNA-based networks described above are often based on noncovalent Watson–Crick interactions between the individual nucleic-acid components, a property that enables predictable sequence-specific recognition and catalytic functionality (e.g., thanks to the use of DNAzymes) and allows different DNA-recognizing enzymes to be used as tools to control either the input or the output of the network.^35−40^ However, despite the above advantages, the use of Watson–Crick interactions also entails an inherent limitation on the overall complexity that these networks can achieve, as each individual DNA-based component can only interact with a limited number of related components via complementary domains.

In nature, however, many naturally occurring circuits or networks consist of groups of components that interact with each other in an all-to-all, many-to-many or promiscuous manner, leading to greater programmability and versatility of the network’s input-output computations.^41^ For example, in a competitive dimerization network, families of monomeric proteins (inputs) compete with each other in various combinations to produce a series of dimer outputs.^42^ Upstream signals or molecular cues can modulate the concentrations of the monomers and thus control the formation of the active dimers downstream. Such dimerization networks are ubiquitous in cells and often regulate genes involved in a variety of processes, including cell proliferation, differentiation and hormone signaling.^43−46^ Motivated by the above considerations, we demonstrate here a DNA-based competitive dimerization network in which, unlike other DNA-based networks, each monomer interacts in an all-to-all or a many-to-many manner through covalent reactions to produce a library of different outputs. The DNA-based competitive dimerization network we describe here takes advantage of the specificity, programmability and orthogonality of DNA–DNA interactions for processing different inputs. We then employ reactive groups conjugated to each DNA monomer strand to generate a library of possible outputs through covalent reactions. By using sequence-specific inputs, this network can then perform complex input-output computation and produce outputs in a highly predictable and programmable manner.

## Results and Discussion

**Figure 1:**
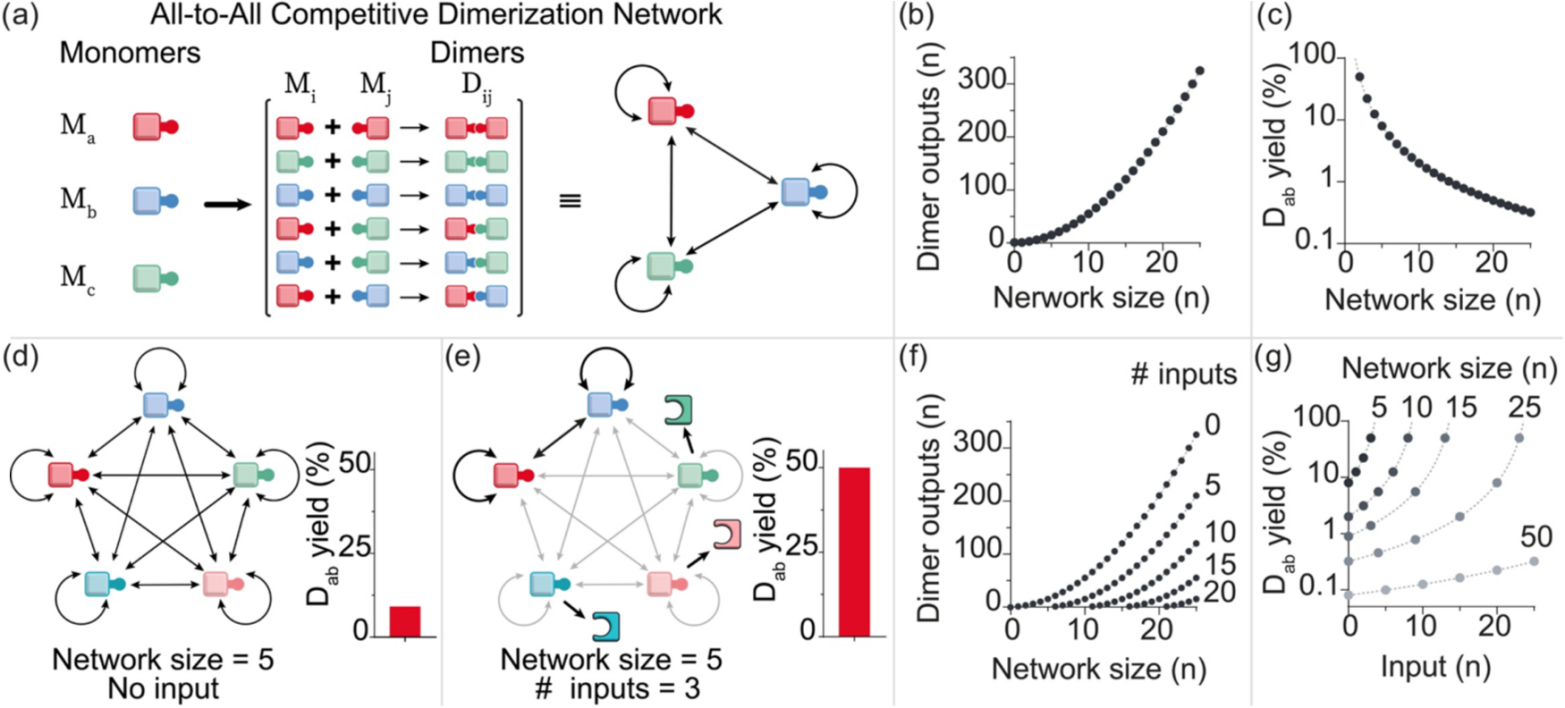
(a) Competitive all-to-all dimerization network consisting of a series of monomers, each of which is capable of reacting with each other to form a library of dimers. (b) The network size (number of different monomers) determines the number of possible dimer outputs and (c) the theoretical yield (%) of a particular dimer output (here the heterodimer D_ab_). (d) An all-to-all dimerization network with 5 monomers leads to an expected yield of the heterodimer output D_ab_ of about 8% (bar chart). (e) The same all-to-all dimerization network (5 monomers) with 3 inputs that sequester 3 monomers from the network leads to an expected yield of the same heterodimer D_ab_ output of about 50%. (f) Diagrams of the number of possible dimer outputs compared to network sizes with different numbers of inputs. (g) Diagrams of the yield of the heterodimer output D_ab_ compared to the number of inputs for a fixed network size (5, 10, 15, 25 and 50).

In this work, we first consider a competitive dimerization network consisting of *m* interacting monomers (M_a_, M_b_, ···, M_m_) that can covalently connect to each other to form a library of dimer outputs (D_ab_, D_ac_, ···, D_mm_) (Figure 1a). Each pair of monomers has the same equilibrium constant for the formation of a dimer and (unless otherwise stated) each monomer has the same concentration, so that random formation of all possible dimer outputs can be expected (i.e., each dimer has a similar statistically determined probability of forming). In this situation, the number of possible dimer outputs increases as the size of the network increases according to the following equation (Figure 1b)
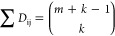
1where *m* is the network size (i.e., the total number of different monomers) and *k* is the output size (i.e., the number of monomers composing each output). So, in case of a dimerization reaction the output size is 2 and eq 1 can be simplified as
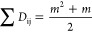
2

As the network size increases, the yield (%) of a specific dimer (defined here as the target dimer output, *D*_ij_) thus decreases according to the following function (Figure 1c)
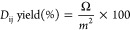
3Where Ω is 1 in case of a homodimer output and 2 in case of a heterodimer output.

The addition of molecular cues (i.e., inputs) that specifically bind and sequester some of the monomers would reduce the overall size of such competitive dimerization network and the number of possible dimer outputs (Figure 1d). Thus, the addition of inputs (in this case we consider a saturating concentration of each input) leads to an increase in the overall yield of the target heterodimer output as described by the following eq (Figure 1e–g)
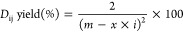
4Where *x* is the number of different monomers excluded by each input, *i*.

**Figure 2:**
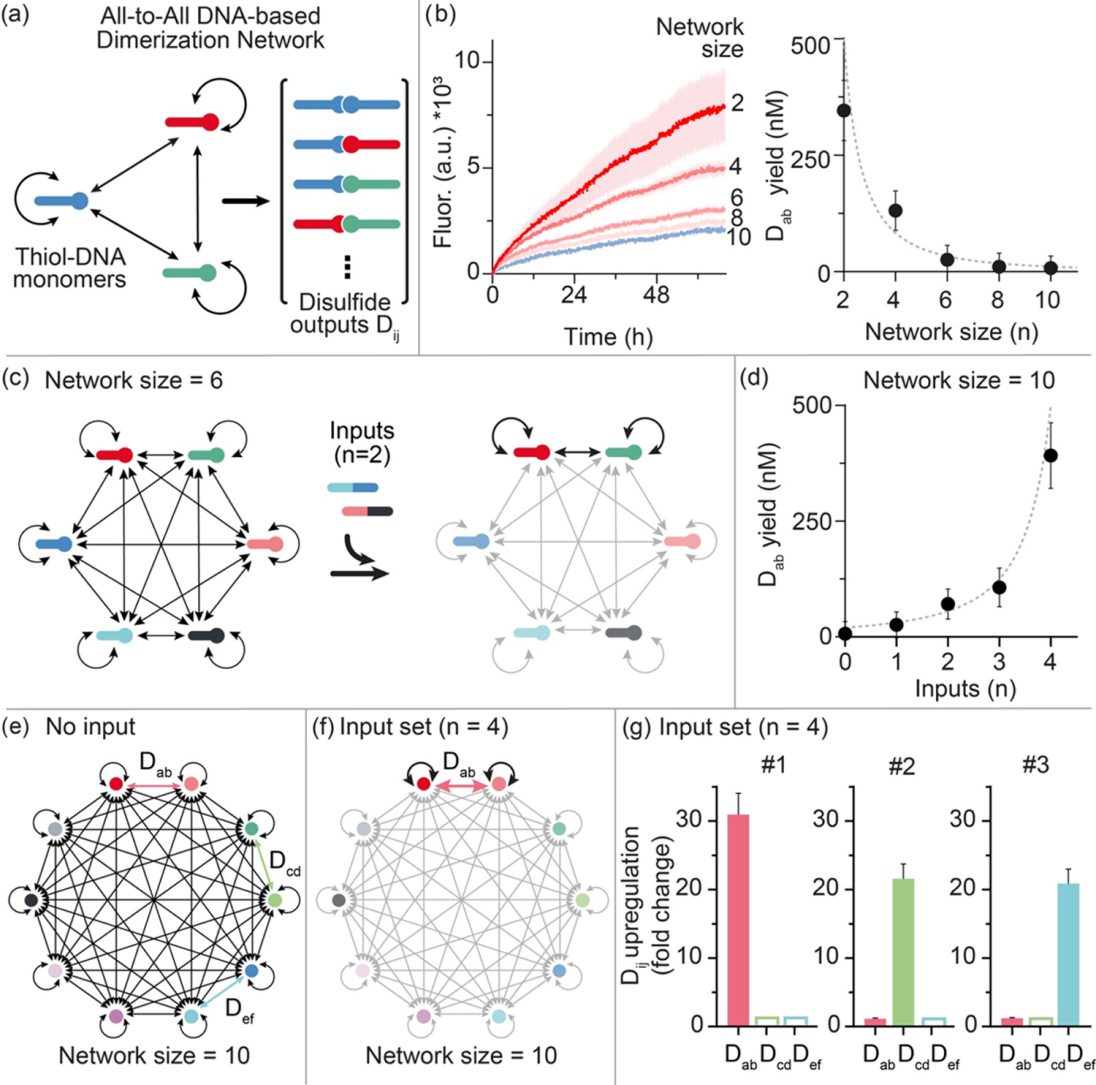
DNA-based dimerization network using disulfide formation. (a) Each monomer of the network is a thiol-modified single-stranded DNA oligonucleotide. The formation of disulfide bonds generates the dimer outputs. (b) (left) Kinetics of the formation of the target dimer output (D_ab_) for different network sizes (2, 4, 6, 8, and 10 monomers); (right) yield of the target dimer output (nM) as a function of network size. The dashed line represents the theoretical yield. (c) The reaction network can be controlled by addition of input strands. The input strands here are designed to hybridize to two monomers and exclude them from the network. The example shows a network of 6 monomers before and after addition of 2 inputs. (d) Yield (nM) of the target dimer (D_ab_) as a function of the number of inputs (network size = 10). The dashed line represents the theoretical yield. (e) Schematic of an all-to-all dimerization network formed by 10 different thiol-DNA monomers. (f) For a fixed network size (*n* = 10), it is possible to use different sets of inputs (each set contains 4 different inputs) to induce upregulation of a different target dimer output. (g) Upregulation of 3 different dimer outputs (D_ab_, D_cd_, D_ef_) using 3 different sets of inputs obtained as the ratio between the yield values calculated in the presence and absence of the indicated input set. The experiments shown in this figure were performed in 1 × TAE buffer, 12.5 mM MgCl_2_, pH 8.5. Each thiol-DNA monomer and each input was used at a concentration of 1.0 μM and the dimerization reaction was started adding 1.0 mM of NaBO_3_. If present, the inputs were added immediately before the start of the dimerization reaction. Reaction mixtures also contain the reporters for quantification of the dimer output yield. Error bars represent the standard deviation based on triplicate measurements.

To establish a DNA-based all-to-all competitive dimerization network, we designed and synthesized a set of noninteracting single-stranded DNA oligonucleotide monomers (length between 8 and 22 nts) (Figure S1), each with a specific sequence and modified with a thiol group (i.e., R–C_6_–SH) either at the 3′-end or at the 5′-end (Figure 2a). Under oxidizing conditions, such a dimerization network can induce the formation of a library of disulfide dimer outputs.

We have identified one of the possible dimer outputs (here the heterodimer D_ab_) as the “target” output. We can measure the formation (and thus the overall yield) of such a target output under different experimental conditions by a strand displacement reaction with an optically labeled DNA duplex (Figures S2 and S3). For example, we performed experiments with DNA-based dimerization networks of different sizes (different number of monomers) using equimolar concentrations of each monomer (i.e., 1.0 μM) and found that the yield of the target dimer D_ab_ decreases from 350 ± 60 to 7 ± 20 nM when we increase the network size from 2 to 10 monomers (Figure 2b). As expected, the observed yield of the target for the different network sizes agrees well with the theoretical yield (Figure 2b, dotted line).

We can rationally control the yield of the dimer output D_ab_ by introducing into our dimerization network different molecular inputs that act as specific sequesters of certain monomers. To this end, we designed and synthesized DNA strands with a sequence that is fully complementary to that of two monomers, such that a single input is able to exclude two monomers from the dimerization network (Figure 2c). With a dimerization network size of 10 (using an equimolar concentration of monomers and inputs of 1.0 μM), by adding 1 to 4 inputs we were able to control the yield of the target dimer D_ab_ from 25 ± 30 to 390 ± 70 nM, respectively (Figure 2d). Also in this case, the observed yield of the dimer output agrees very well with the expected yield under each experimental condition used (Figure 2d, dotted line). The system can respond to inputs in a highly dynamic fashion. To demonstrate this, we used the same DNA-based dimerization network employed above (*n* = 10) and we added 4 inputs at different times. By doing so, we were able to finely modulate the relative yield of the dimer output D_ab_ between 360 ± 60 and 60 ± 30 nM by increasing the time of addition of the inputs from 0 to 24 h. (Figure S4).

The network size can also be controlled by tuning the concentration of each monomer in the reaction mixture. To demonstrate this, we prepared a dimerization network with 4 different thiol-DNA monomers in which two monomers (M_c_, M_d_) display a 4-fold higher concentration compared to the two output-forming monomers (M_a_, M_b_). Under these conditions the expected yield of D_ab_ will be the same as that expected in a network size of 10 monomers under equimolar conditions. We can control the yield of the dimer output D_ab_ by varying the concentration of the single input sequestering the two nonfunctional monomers. More specifically, by increasing the input concentration from 0.5 to 10 μM we were able to increase the yield of the target dimer D_ab_ from 8 ± 10 to 270 ± 30 nM (Figure S5).

Our DNA-based dimerization network allows to achieve highly programmable input-output computation by the rational design of different sets of inputs. To this end, we employed the same dimerization network (size = 10) described before (Figure 2d) and we selected 3 different target heterodimer outputs (i.e., D_ab_, D_cd_, D_ef_). We then synthesized 3 different sets of inputs (each set displaying 4 inputs) to induce the controlled upregulation of such dimer outputs in the same dimerization network (Figure 2e–f). The upregulation is specific and orthogonal, so a different upregulated dimer output can be achieved by simply changing the input set (Figure 2g). We can also upregulate in the same solution two different dimer outputs, although with a slightly lower efficiency, by reducing the number of inputs (i.e., 3) in each input set (Figure S6). Similar orthogonal and programmable upregulation of different dimer outputs using different sets of inputs can also be achieved with larger network size (i.e., 30 monomers) providing a further demonstration of the computational ability of such DNA-based dimerization networks (Figure S7).

**Figure 3:**
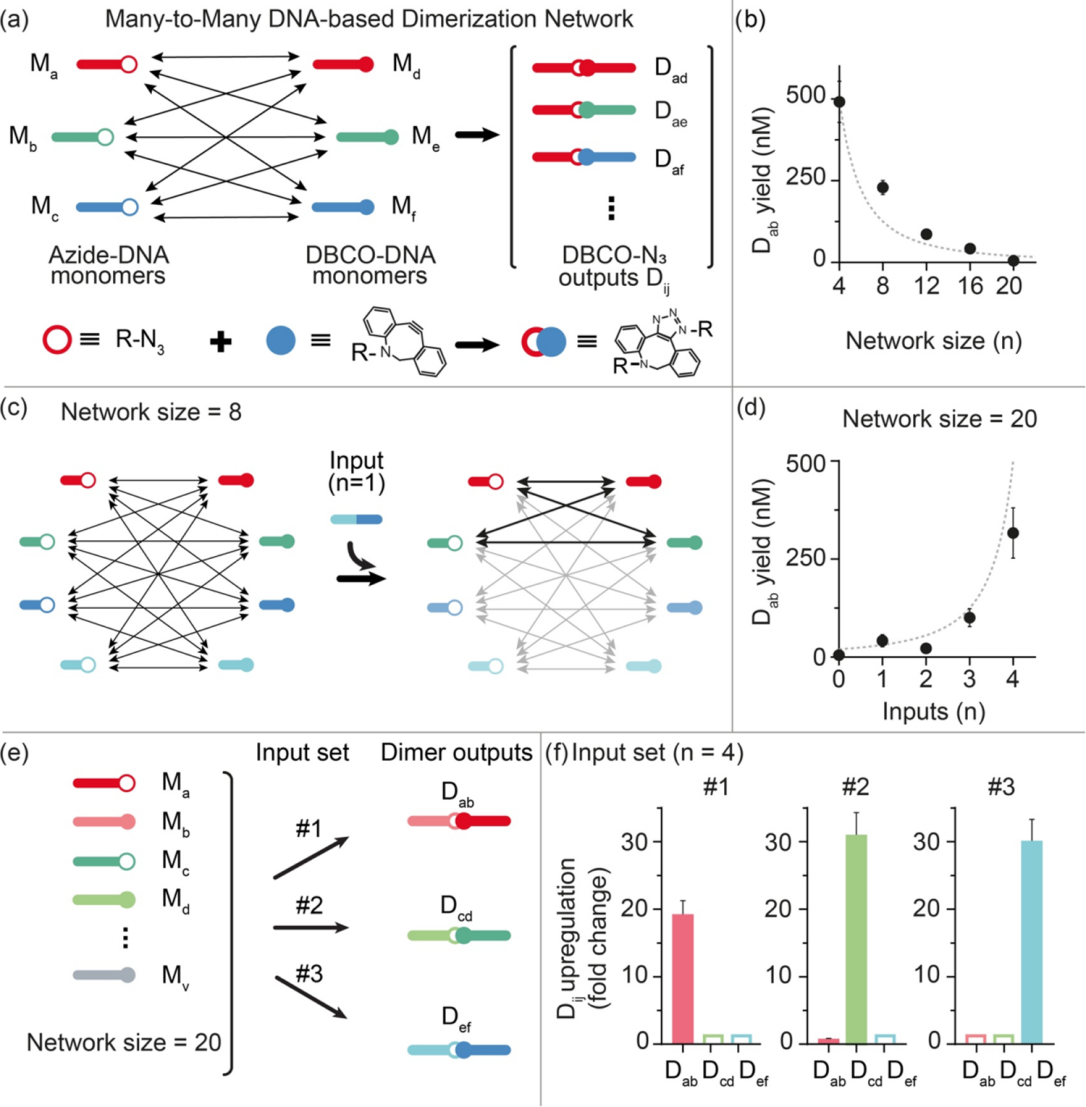
DNA-based dimerization network using SPAAC reactions. (a) Each monomer of the network is a single-stranded DNA oligonucleotide modified with either azide or DBCO. The formation of the DBCO-azide conjugate generates the dimer outputs. (b) Yield of dimer outputs (nM) as a function of network size. The dashed line represents the theoretical yield. (c) The reaction network can be controlled by addition of input strands. The input strands here are designed to hybridize to 4 monomers and exclude them from the network. The example shows a network of 8 monomers before and after addition of 1 input. (d) Yield of target dimers (nM) as a function of the number of inputs. The dashed line represents the theoretical yield. Here the network size is 20. (e) With a fixed network size (*n* = 20), it is possible to use different sets of inputs (4 different inputs in each set) to induce upregulation of a different target dimer output. (f) Upregulation of 3 different dimer outputs (D_ab_, D_cd_, D_ef_) after addition of 3 different sets of inputs obtained as the ratio between the yield values calculated in the presence and absence of the indicated input set. The experiments shown in this figure were performed in carbonate buffer 50 mM NaHCO_3_, 1.0 M NaCl, pH 8.6. Each azide- and DBCO–DNA monomer was mixed at a concentration of 0.5 μM. If present, inputs were used at a concentration of 1.0 μM and were introduced to the DBCO–DNA monomers immediately before adding the azide-DNA monomers. Error bars represent the standard deviation based on triplicate measurements.

To demonstrate the versatility of DNA-based dimerization networks, we designed and synthesized new modified DNA sequences to create a many-to-many dimerization network that employs a different chemical reaction. More specifically, we synthesized ss-DNA monomers modified with either a dibenzocyclooctyne (DBCO) group or an azide group at one of the two ends of the strands, such that a spontaneous and irreversible strain-promoted azide–alkyne cycloaddition (SPAAC) reaction between these two reactive groups would lead to a dimer output formed by an azide-DBCO conjugate (Figure 3a). In this case, the formation of homodimers is not possible, so the total number of possible interactions is reduced and the dependence of the number of possible dimer outputs on the size of the dimerization network follows the equation below
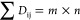
5Where *m* is the number of monomers modified with DBCO and *n* is the number of monomers modified with azide.

Also in this case, the yield of a selected target dimer (D_ab_) was measured across different network sizes with a specific strand displacement reaction, and the experimental results are in good agreement with the expected yield under each tested condition (Figure 3b). We can modulate and control the yield of the target dimer output by introducing DNA inputs that, by binding to specific monomers, exclude them from the network and thus upregulate the formation of the target dimer output (Figure 3c). To demonstrate this, we designed a network with 20 monomers (10 modified with DBCO and 10 with azide) (Figure 3d) and we carried out reactions after addition of different input strands. By doing so, we were able to upregulate the D_ab_ dimer output from 5 ± 20 nM (no input) to 320 ± 20 nM (4 inputs) (Figure 3d). Also in this case, the input-output behavior of this DNA-based dimerization network is highly programmable, so that the formation of different target dimer outputs can be upregulated by different input sets (Figure 3e,f). We were also able to upregulate in the same solution two different dimer outputs by reducing the number of inputs (i.e., 3) in each input set (Figure S8).

**Figure 4:**
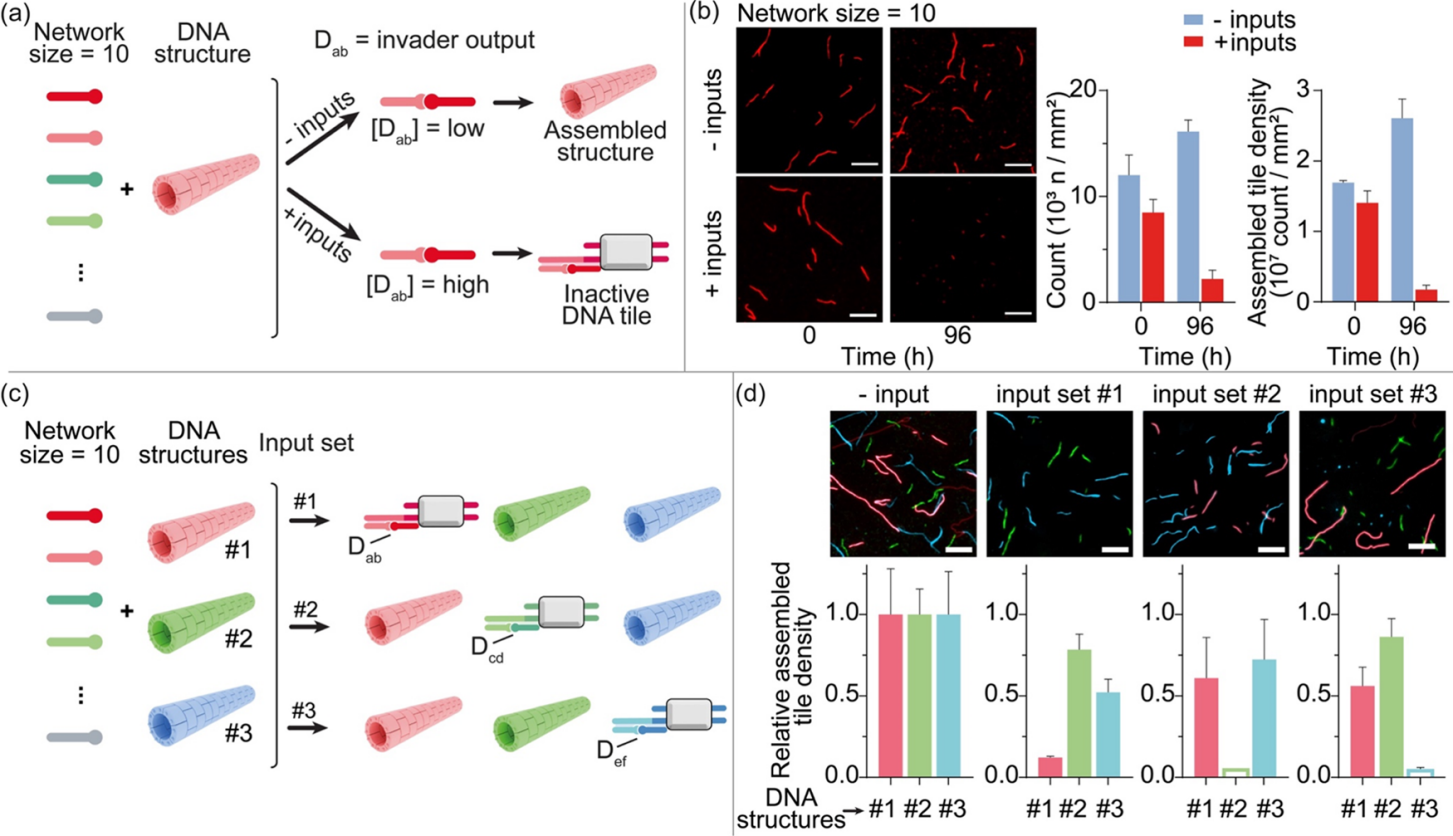
DNA dimerization networks for the control of DNA structures. (a) Schematic representation of a dimerization network of thiol-DNA monomers that produces, among other outputs, a dimer (D_ab_) that induces the disassembly of a DNA structure. Only in the presence of inputs, such an invader output is upregulated and DNA structures disassembled. (b) Fluorescence images and histograms showing the count and the density of assembled tiles obtained with the dimerization network (size = 10) in the absence and presence of inputs (at 0 and 96 h). (c) Upregulation of three different dimer invader outputs in the same dimerization network (*n* = 10) using three different sets of inputs in a mixture containing three different DNA structures (#1, #2, #3). Each dimer invader output is designed to specifically induce disassembly of only one of the three DNA structures. (d) Fluorescence images and relative density of assembled tiles for each of the three structures obtained with the dimerization network (size = 10) in the absence and presence of the three sets of inputs (at 72 h). The experiments shown in this figure were performed in 1 × TAE buffer, 12.5 mM MgCl_2_, pH 8.5. Each thiol-DNA monomer and each input was used at a concentration of 1.0 μM. The DNA structures were used at a concentration of DNA tiles of 100 nM and the dimerization reaction was started adding 1.0 mM of NaBO_3_. Error bars represent the standard deviation based on triplicate measurements.

Next, we tested whether our DNA-based dimerization network can be used to control downstream reaction pathways. To do this, we used an all-to-all dimerization network with 10 thiol-modified DNA monomers similar to that shown in Figure 2. We designed two of the monomers so that their dimerization generates an output strand that can trigger the downstream disassembly of a DNA-based nanostructure (Figure 4a). More specifically, we used as DNA nanostructure a tubular object formed by the self-assembly of DNA “tiles” through hybridization of their complementary “sticky ends”.^47^ These structures self-assemble at room temperature and can be disassembled by introducing a DNA strand (invader) that binds to the tiles and “invade” the sticky end (Figure S9).^47,48^ This type of assembly and disassembly mechanism can be easily monitored by labeling a tile-forming strand with a fluorophore so that the DNA structure can be visualized by fluorescence microscopy. Under competitive dimerization conditions, our dimerization network generates a dimer invader concentration (i.e., 40 ± 10 nM) that is not sufficient to observe significant disassembly of DNA structures (considering that the concentration of DNA tiles in solution is 100 nM) (Figure 4b). This is consistent with control experiments showing that under the experimental conditions used, a minimum concentration of 300 nM of invader DNA strand is required to observe disassembly of the DNA structures (Figure S10). Only with the addition of the set of input strands (*n* = 4) required to upregulate the dimer invader strand, can the disassembly of the DNA tubes be observed over time (Figure 4b). The successful disassembly is demonstrated both by the reduced number of DNA structures (i.e., defined here as count per mm^2^), which changes from 8 ± 1 to 2.0 ± 0.6 in the presence of the input set, and the reduced density of assembled tiles (from 1.4 ± 0.2 to 0.18 ± 0.06 × 10^7^ count/mm^2^) (Figure 4b).

The versatility of the DNA dimerization network is again demonstrated by using the same network (size = 10) with three different input sets to allow orthogonal upregulation of three different output dimer invaders, each capable of specifically disassembling a different DNA structure (Figure 4c). To this end, we first designed and characterized three DNA-based structures, each formed by the self-assembly of three orthogonal DNA tiles. To allow easy characterization of these structures, each tile was labeled with a different fluorophore with nonoverlapping emission and excitation wavelengths. Each of these structures can be disassembled by a specific dimer output invader strand (i.e., D_ab_, D_cd_, D_ef_). By introducing the different inputs into the dimerization network, we were able to upregulate only one of the three specific dimer invaders and thus disassemble one of the three coexisting DNA structures in solution (Figure 4d).

## Conclusions

It is known that most cellular metabolic pathways consist of simple elements that interact with each other and can process input information (encoded as molecular or environmental signals) in a highly flexible and complex manner.^44,50^ This type of input-output computation is very common in cells and can provide higher level functions in signal transduction,^50,51^ adhesion^52,53^ and transcriptional regulation.^54,55^ One of the most intriguing of these computational mechanisms, namely protein competitive dimerization, involves a set of proteins that are able to interact with each other in a many-to-many or an all-to-all manner. Such protein-based promiscuous networks can provide powerful computational capabilities that are particularly crucial in multicellular organisms,^43,56^ and their importance in natural biological contexts is currently being uncovered.^42^

Inspired by this mechanism, we demonstrate here a synthetic DNA-based dimerization network consisting of a series of DNA oligonucleotides modified with reactive moieties that can covalently bind to each other either in an all-to-all or in a many-to-many fashion. In this way, we demonstrate that highly programmable input-output computation can be achieved, allowing the yield of a given DNA dimer output to be controlled in a predictable manner. We have also shown that the size of the DNA-based competitive dimerization network can be scaled up in a quite straightforward manner thus allowing to achieve higher computational capabilities of the system.^42^ Finally, DNA-based dimerization networks were used to modulate the yield of a functional dimer output to ultimately control the assembly and disassembly of synthetic DNA nanostructures. And although the DNA-based dimerization networks we introduce here are not reversible as the protein-based naturally occurring counterparts, they display flexible and dynamic behavior.

Compared to other examples of DNA-based networks and circuits where hybridization of base pairs drives both input recognition and output formation,^26−35^ here we present an alternative strategy that could improve the computational capability of these systems. Our approach exploits the predictability of DNA–DNA interactions to achieve specific input/output computation. However, unlike previously demonstrated DNA networks, it utilizes covalent reactions between the DNA-based reactive units of the network to create a larger chemical space for output generation. Combining the programmability of DNA hybridization with the ability to explore different reactive functionalizations of DNA strands could provide a simple route to developing more complex promiscuous architectures and networks that lead to a broader range of functions and can be used to develop synthetic systems that mimic functions of living cells and respond to molecular cues to generate specific signals. This can find sensing, diagnostic or therapeutic applications. For example, it would be interesting to create DNA-based dimerization networks similar to those shown here, where the DNA output is capable of controlling a relevant biochemical pathway. Given the central role of DNA in genetic circuits, the most obvious application of a similar network could also be the development of synthetic genetic networks capable of converting specific inputs into the expression of an output protein. Similar input/output computational mechanisms can be also designed to produce higher-order structures (trimers, tetramers, etc.) that can be used, for example, to achieve input-controlled programmable recombination in synthetic templates.

## Experimental Section

### Chemicals

All reagent-grade chemicals, including MgCl_2_, Trizma Base, ethylenediaminetetraacetic acid (EDTA), NaCl, sodium bicarbonate NaHCO_3_, tris(2-carboxyethyl) phosphine hydrochloride (TCEP) and sodium perborate (NaBO_3_**·**4H_2_O) were purchased from Sigma-Aldrich (Italy) and used without further purification.

### Oligonucleotides

Oligonucleotides employed in this work were synthesized, labeled, and high-performance liquid chromatography (HPLC)-purified by Metabion International AG (Planegg, Germany) and used without further purification. The DNA oligonucleotides were dissolved in phosphate buffer (50 mM, pH 7.0) and stored at −20 °C until use. All the sequences of the different systems are reported in the Supporting Information.

### DNA-Based Dimerization Network Using Disulfide Formation

To ensure the absence of unnecessary thiol groups in the samples, disulfide-DNA dimers were used to generate thiolated-DNA monomers. Each disulfide-DNA dimer (20 μM) was reduced overnight with a solution of 1.0 mM TCEP, prepared in 1 × TAE buffer (i.e., 40 mM Tris base + 40 mM acetate + 1 mM EDTA, pH 8.5) + 12.5 mM MgCl_2_, at room temperature, to allow quantitative reduction of disulfide bonds. After reduction, the so-obtained thiolated-DNA monomers were mixed and diluted to a final concentration of 1.0 μM in 1 × TAE buffer + 12.5 mM MgCl_2_, pH 8.5. The dimerization process is started by adding an oxidizing agent to the sample (1.0 mM NaBO_3_). If inputs are used (Figure 2d,g) they are added immediately before the oxidizing agent. Both TCEP and NaBO_3_ were freshly prepared before use.

### DNA-Based Dimerization Network Using SPAAC Reactions

The dimerization reaction is carried out at room temperature using a bicarbonate buffer (50 mM NaHCO_3_, 1.0 M NaCl, pH 8.6). Two separate solutions, each containing 1.0 μM of DBCO–DNA monomers and azide-DNA monomers, are prepared in the same bicarbonate buffer. Equal volumes of these solutions are then mixed in a 1:1 ratio to initiate the SPAAC reaction so that the final concentration of each azide- and DBCO-monomer is 0.5 μM. If inputs are used (Figure 3d,3f) they are added to the DBCO–DNA monomers immediately before adding the azide-DNA monomers.

### Fluorescence Experiments

Fluorescence kinetic measurements were carried out on a Tecan F200pro plate reader using the top reading mode with black, flat bottom nonbinding 384-well plates and a 30 μL final volume. Detailed procedures employed are reported in the Supporting Information.

### Self-Assembly of DNA Nanostructures

The tile design and sequences employed in this study are described elsewhere.^47−49^ Briefly, DNA tiles for all the systems were prepared as follows: tile-forming strands were mixed at a final concentration of 5.0 μM in H_2_O/Mg^2+^ (12.5 mM MgCl_2_), and annealed using a thermocycler (Bio-Rad T100 thermal cycler) by heating the solution to 90 °C and cooling it to 20 °C at a constant rate for a 6 h period. The concentrations employed and buffer conditions for DNA nanostructure disassembly are reported in the caption of the corresponding figure and in the Supporting Information.

### Fluorescence Imaging of DNA-Based Nanostructures

An Axio Observer 7 ZEISS microscope was used for fluorescence microscopy imaging. The images were acquired with a 100× oil objective and a monochrome CCD camera (Axiocam 305 mono-ZEISS). Images were analyzed and processed to correct for uneven illumination and superimposed to produce multicolor images using ZEN-3.3 lite (ZEISS) software. Average length and count of assembled scaffolds were quantified by image metrology using SPIP software.

## Supporting Information Available

Oligonucleotide sequences used; image analysis protocols and supporting figures (PDF)

## References

[ref1] Ashkenasy G.; Hermans T. M.; Otto S.; Taylor A. F. Systems Chemistry. Chem. Soc. Rev. 2017, 46 (9), 2543–2554. 10.1039/C7CS00117G. 28418049

[ref2] Mattia E.; Otto S. Supramolecular Systems Chemistry. Nat. Nanotechnol. 2015, 10 (2), 111–119. 10.1038/nnano.2014.337. 25652169

[ref3] Ludlow R. F.; Otto S. Systems Chemistry. Chem. Soc. Rev. 2008, 37 (1), 101–108. 10.1039/B611921M. 18197336

[ref4] Miyata M.; Robinson R. C.; Uyeda T. Q. P.; Fukumori Y.; Fukushima S.; Haruta S.; Homma M.; Inaba K.; Ito M.; Kaito C.; Kato K.; Kenri T.; Kinosita Y.; Kojima S.; Minamino T.; Mori H.; Nakamura S.; Nakane D.; Nakayama K.; Nishiyama M.; Shibata S.; Shimabukuro K.; Tamakoshi M.; Taoka A.; Tashiro Y.; Tulum I.; Wada H.; Wakabayashi K. Tree of Motility – A Proposed History of Motility Systems in the Tree of Life. Genes Cells 2020, 25 (1), 6–21. 10.1111/gtc.12737. 31957229 PMC7004002

[ref5] Wang L.; Song S.; van Hest J.; Abdelmohsen L. K. E. A.; Huang X.; Sánchez S. Biomimicry of Cellular Motility and Communication Based on Synthetic Soft-Architectures. Small 2020, 16, e1907680 10.1002/smll.201907680. 32250035

[ref6] Marincioni B.; Nakashima K. K.; Katsonis N. Motility of Microscopic Swimmers as Protocells. Chem 2023, 9 (11), 3030–3044. 10.1016/j.chempr.2023.10.007.

[ref7] Vay K. L.; Weise L. I.; Libicher K.; Mascarenhas J.; Mutschler H. Templated Self-Replication in Biomimetic Systems. Adv. Biosyst. 2019, 3 (6), e1800313 10.1002/adbi.201800313. 32648707

[ref8] Duim H.; Otto S. Towards Open-Ended Evolution in Self-Replicating Molecular Systems. Beilstein J. Org. Chem. 2017, 13, 1189–1203. 10.3762/bjoc.13.118. 28694865 PMC5496545

[ref9] Vidonne A.; Philp D. Making Molecules Make Themselves – the Chemistry of Artificial Replicators. Eur. J. Org. Chem. 2009, 2009, 593–610. 10.1002/ejoc.200800827.

[ref10] Liu K.; Blokhuis A.; van Ewijk C.; Kiani A.; Wu J.; Roos W. H.; Otto S. Light-Driven Eco-Evolutionary Dynamics in a Synthetic Replicator System. Nat. Chem. 2024, 16 (1), 79–88. 10.1038/s41557-023-01301-2. 37653230

[ref11] Eleveld M. J.; Geiger Y.; Wu J.; Kiani A.; Schaeffer G.; Otto S. Competitive Exclusion among Self-Replicating Molecules Curtails the Tendency of Chemistry to Diversify. Nat. Chem. 2025, 17, 132–140. 10.1038/s41557-024-01664-0. 39613869

[ref12] Lauber N.; Flamm C.; Ruiz-Mirazo K. Minimal Metabolism”: A Key Concept to Investigate the Origins and Nature of Biological Systems. BioEssays 2021, 43 (10), e2100103 10.1002/bies.202100103. 34426986

[ref13] Muchowska K. B.; Varma S. J.; Moran J. Nonenzymatic Metabolic Reactions and Life’s Origins. Chem. Rev. 2020, 120 (15), 7708–7744. 10.1021/acs.chemrev.0c00191. 32687326

[ref14] Corbett P. T.; Leclaire J.; Vial L.; West K. R.; Wietor J.-L.; Sanders J. K. M.; Otto S. Dynamic Combinatorial Chemistry. Chem. Rev. 2006, 106 (9), 3652–3711. 10.1021/cr020452p. 16967917

[ref15] Jia C.; Qi D.; Zhang Y.; Rissanen K.; Li J. Strategies for Exploring Functions from Dynamic Combinatorial Libraries. ChemSystemsChem 2020, 2 (5), e2000019 10.1002/syst.202000019.

[ref16] Jin Y.; Yu C.; Denman R. J.; Zhang W. Recent Advances in Dynamic Covalent Chemistry. Chem. Soc. Rev. 2013, 42 (16), 6634–6654. 10.1039/c3cs60044k. 23749182

[ref17] Corbett P. T.; Sanders J. K. M.; Otto S. Systems Chemistry: Pattern Formation in Random Dynamic Combinatorial Libraries. Angew. Chem., Int. Ed. 2007, 46 (46), 8858–8861. 10.1002/anie.200702460. 17943931

[ref18] Osypenko A.; Dhers S.; Lehn J.-M. Pattern Generation and Information Transfer through a Liquid/Liquid Interface in 3D Constitutional Dynamic Networks of Imine Ligands in Response to Metal Cation Effectors. J. Am. Chem. Soc. 2019, 141 (32), 12724–12737. 10.1021/jacs.9b05438. 31364844

[ref19] Men G.; Lehn J.-M. Multiple Adaptation of Constitutional Dynamic Networks and Information Storage in Constitutional Distributions of Acylhydrazones. Chem. Sci. 2019, 10 (1), 90–98. 10.1039/C8SC03858A. 30713621 PMC6333171

[ref20] Holub J.; Vantomme G.; Lehn J.-M. Training a Constitutional Dynamic Network for Effector Recognition: Storage, Recall, and Erasing of Information. J. Am. Chem. Soc. 2016, 138 (36), 11783–11791. 10.1021/jacs.6b05785. 27571554

[ref21] Lehn J.-M. From Supramolecular Chemistry towards Constitutional Dynamic Chemistry and Adaptive Chemistry. Chem. Soc. Rev. 2007, 36 (2), 151–160. 10.1039/B616752G. 17264919

[ref22] Schaffter S. W.; Schulman R. Building in Vitro Transcriptional Regulatory Networks by Successively Integrating Multiple Functional Circuit Modules. Nat. Chem. 2019, 11 (9), 829–838. 10.1038/s41557-019-0292-z. 31427767

[ref23] Li J.; Green A. A.; Yan H.; Fan C. Engineering Nucleic Acid Structures for Programmable Molecular Circuitry and Intracellular Biocomputation. Nat. Chem. 2017, 9 (11), 1056–1067. 10.1038/nchem.2852. 29064489 PMC11421837

[ref24] Sharma C.; Samanta A.; Schmidt R. S.; Walther A. DNA-Based Signaling Networks for Transient Colloidal Co-Assemblies. J. Am. Chem. Soc. 2023, 145 (32), 17819–17830. 10.1021/jacs.3c04807. 37543962

[ref25] Oesinghaus L.; Simmel F. C. Switching the Activity of Cas12a Using Guide RNA Strand Displacement Circuits. Nat. Commun. 2019, 10 (1), 2092 10.1038/s41467-019-09953-w. 31064995 PMC6504869

[ref26] Qian L.; Winfree E. Scaling Up Digital Circuit Computation with DNA Strand Displacement Cascades. Science 2011, 332 (6034), 1196–1201. 10.1126/science.1200520. 21636773

[ref27] Yang S.; Bögels B. W. A.; Wang F.; Xu C.; Dou H.; Mann S.; Fan C.; de Greef T. F. A. DNA as a Universal Chemical Substrate for Computing and Data Storage. Nat. Rev. Chem. 2024, 8 (3), 179–194. 10.1038/s41570-024-00576-4. 38337008

[ref28] Wang F.; Lv H.; Li Q.; Li J.; Zhang X.; Shi J.; Wang L.; Fan C. Implementing Digital Computing with DNA-Based Switching Circuits. Nat. Commun. 2020, 11 (1), 121 10.1038/s41467-019-13980-y. 31913309 PMC6949259

[ref29] Gong J.; Tsumura N.; Sato Y.; Takinoue M. Computational DNA Droplets Recognizing MiRNA Sequence Inputs Based on Liquid–Liquid Phase Separation. Adv. Funct. Mater. 2022, 32 (37), 2202322 10.1002/adfm.202202322.

[ref30] Cherry K. M.; Qian L. Scaling up Molecular Pattern Recognition with DNA-Based Winner-Take-All Neural Networks. Nature 2018, 559 (7714), 370–376. 10.1038/s41586-018-0289-6. 29973727

[ref31] Kieffer C.; Genot A. J.; Rondelez Y.; Gines G. Molecular Computation for Molecular Classification. Adv. Biol. 2023, 7 (3), 2200203 10.1002/adbi.202200203. 36709492

[ref32] Joesaar A.; Yang S.; Bögels B.; van der Linden A.; Pieters P.; Kumar B. V. V. S. P.; Dalchau N.; Phillips A.; Mann S.; de Greef T. F. A. DNA-Based Communication in Populations of Synthetic Protocells. Nat. Nanotechnol. 2019, 14 (4), 369–378. 10.1038/s41565-019-0399-9. 30833694 PMC6451639

[ref33] Deng J.; Walther A. Autonomous DNA Nanostructures Instructed by Hierarchically Concatenated Chemical Reaction Networks. Nat. Commun. 2021, 12 (1), 5132 10.1038/s41467-021-25450-5. 34446724 PMC8390752

[ref34] Weitz M.; Kim J.; Kapsner K.; Winfree E.; Franco E.; Simmel F. C. Diversity in the dynamical behaviour of a compartmentalized programmable biochemical oscillator. Nat. Chem. 2014, 6, 295–302. 10.1038/nchem.1869. 24651195

[ref35] Wang S.; Yue L.; Shpilt Z.; Cecconello A.; Kahn J. S.; Lehn J.-M.; Willner I. Controlling the Catalytic Functions of DNAzymes within Constitutional Dynamic Networks of DNA Nanostructures. J. Am. Chem. Soc. 2017, 139 (28), 9662–9671. 10.1021/jacs.7b04531. 28627887

[ref36] Yue L.; Wang S.; Lilienthal S.; Wulf V.; Remacle F.; Levine R. D.; Willner I. Intercommunication of DNA-Based Constitutional Dynamic Networks. J. Am. Chem. Soc. 2018, 140 (28), 8721–8731. 10.1021/jacs.8b03450. 29965742

[ref37] Yue L.; Wang S.; Wulf V.; Lilienthal S.; Remacle F.; Levine R. D.; Willner I. Consecutive Feedback-Driven Constitutional Dynamic Networks. Proc. Natl. Acad. Sci. U.S.A. 2019, 116 (8), 2843–2848. 10.1073/pnas.1816670116. 30728303 PMC6386722

[ref38] Yue L.; Wang S.; Willner I. Three-Dimensional Nucleic-Acid-Based Constitutional Dynamic Networks: Enhancing Diversity through Complexity of the Systems. J. Am. Chem. Soc. 2019, 141 (41), 16461–16470. 10.1021/jacs.9b08709. 31539236

[ref39] Zhou Z.; Zhang P.; Yue L.; Willner I. Triggered Interconversion of Dynamic Networks Composed of DNA-Tetrahedra Nanostructures. Nano Lett. 2019, 19 (10), 7540–7547. 10.1021/acs.nanolett.9b03606. 31549514

[ref40] Yue L.; Wang S.; Willner I. Triggered Reversible Substitution of Adaptive Constitutional Dynamic Networks Dictates Programmed Catalytic Functions. Sci. Adv. 2019, 5 (5), eaav5564 10.1126/sciadv.aav5564. 31093526 PMC6510552

[ref41] Su C. J.; Murugan A.; Linton J. M.; Yeluri A.; Bois J.; Klumpe H.; Langley M. A.; Antebi Y. E.; Elowitz M. B. Ligand-Receptor Promiscuity Enables Cellular Addressing. Cell Syst. 2022, 13 (5), 408–425.e12. 10.1016/j.cels.2022.03.001. 35421362 PMC10897978

[ref42] Parres-Gold J.; Levine M.; Emert B.; Stuart A.; Elowitz M. B. Principles of Computation by Competitive Protein Dimerization Networks BioRxiv, 2023. https://www.biorxiv.org/content/10.1101/2023.10.30.564854v1. (accessed January, 8, 2025). 10.1016/j.cell.2025.01.036 PMC11973712 39978343

[ref43] Granados A. A.; Kanrar N.; Elowitz M. B. Combinatorial Expression Motifs in Signaling Pathways. Cell Genomics 2024, 4 (1), 100463 10.1016/j.xgen.2023.100463. 38216284 PMC10794782

[ref44] Kramer B. A.; del Castillo J. S.; Pelkmans L. Multimodal Perception Links Cellular State to Decision-Making in Single Cells. Science 2022, 377 (6606), 642–648. 10.1126/science.abf4062. 35857483

[ref45] Hidaka R.; Miyazaki K.; Miyazaki M. The E-Id Axis Instructs Adaptive Versus Innate Lineage Cell Fate Choice and Instructs Regulatory T Cell Differentiation. Front. Immunol. 2022, 13, 890056 10.3389/fimmu.2022.890056. 35603170 PMC9120639

[ref46] Klumpe H. E.; Garcia-Ojalvo J.; Elowitz M. B.; Antebi Y. E. The Computational Capabilities of Many-to-Many Protein Interaction Networks. Cell Syst. 2023, 14 (6), 430–446. 10.1016/j.cels.2023.05.001. 37348461 PMC10318606

[ref47] Rothemund P. W. K.; Ekani-Nkodo A.; Papadakis N.; Kumar A.; Fygenson D. K.; Winfree E. Design and Characterization of Programmable DNA Nanotubes. J. Am. Chem. Soc. 2004, 126 (50), 16344–16352. 10.1021/ja044319l. 15600335

[ref48] Green L. N.; Subramanian H. K. K.; Mardanlou V.; Kim J.; Hariadi R. F.; Franco E. Autonomous Dynamic Control of DNA Nanostructure Self-Assembly. Nat. Chem. 2019, 11 (6), 510–520. 10.1038/s41557-019-0251-8. 31011170

[ref49] Gentile S.; Grosso E. D.; Pungchai P. E.; Franco E.; Prins L. J.; Ricci F. Spontaneous Reorganization of DNA-Based Polymers in Higher Ordered Structures Fueled by RNA. J. Am. Chem. Soc. 2021, 143 (48), 20296–20301. 10.1021/jacs.1c09503. 34843256 PMC8662731

[ref50] Antebi Y. E.; Linton J. M.; Klumpe H.; Bintu B.; Gong M.; Su C.; McCardell R.; Elowitz M. B. Combinatorial Signal Perception in the BMP Pathway. Cell 2017, 170 (6), 1184–1196.e24. 10.1016/j.cell.2017.08.015. 28886385 PMC5612783

[ref51] Klumpe H. E.; Langley M. A.; Linton J. M.; Su C. J.; Antebi Y. E.; Elowitz M. B. The Context-Dependent, Combinatorial Logic of BMP Signaling. Cell Syst. 2022, 13 (5), 388–407.e10. 10.1016/j.cels.2022.03.002. 35421361 PMC9127470

[ref52] Sprinzak D.; Lakhanpal A.; LeBon L.; Santat L. A.; Fontes M. E.; Anderson G. A.; Garcia-Ojalvo J.; Elowitz M. B. Cis-Interactions between Notch and Delta Generate Mutually Exclusive Signalling States. Nature 2010, 465 (7294), 86–90. 10.1038/nature08959. 20418862 PMC2886601

[ref53] Schreiner D.; Weiner J. A. Combinatorial Homophilic Interaction between γ-Protocadherin Multimers Greatly Expands the Molecular Diversity of Cell Adhesion. Proc. Natl. Acad. Sci. U.S.A. 2010, 107 (33), 14893–14898. 10.1073/pnas.1004526107. 20679223 PMC2930437

[ref54] Weingarten-Gabbay S.; Segal E. The Grammar of Transcriptional Regulation. Hum. Genet. 2014, 133 (6), 701–711. 10.1007/s00439-013-1413-1. 24390306 PMC4024068

[ref55] Lee T. I.; Young R. A. Transcriptional Regulation and Its Misregulation in Disease. Cell 2013, 152 (6), 1237–1251. 10.1016/j.cell.2013.02.014. 23498934 PMC3640494

[ref56] Chen Z.; Elowitz M. B. Programmable Protein Circuit Design. Cell 2021, 184 (9), 2284–2301. 10.1016/j.cell.2021.03.007. 33848464 PMC8087657

