# Supplementary material for: Covalent Dynamic DNA Networks to Translate Multiple Inputs into Programmable Outputs
Source: J Am Chem Soc. 2025 Feb 5;147(7):5755–63. doi: 10.1021/jacs.4c13854 (PMC11848822; doi:10.1021/jacs.4c13854)
Supplement: Supplementary file 1 — ja4c13854_si_001.pdf [file ja4c13854_si_001.pdf]

# Covalent dynamic DNA networks to translate multiple inputs into programmable outputs

***Simone Brannetti,[1] Serena Gentile,[1,2] Erica Del Grosso[1], Sijbren Otto[3], Francesco Ricci[1,*]***

*[1] Department of Chemical Sciences and Technologies, University of Rome, Tor Vergata, Via della Ricerca Scientifica, 00133 Rome, Italy*

*[2] Pediatric Oncology Unit, Fondazione Policlinico Universitario Agostino Gemelli IRCCS, 00168 Rome, Italy (current address)*

*[3] Centre for Systems Chemistry, Stratingh Institute, University of Groningen, Groningen 9747 AG, The Netherlands*

** Corresponding author: francesco.ricci@uniroma2.it*

# Supporting information

## 1. DNA sequences

### 1.1. DNA-based dimerization network by disulfide formation

The sequences of the thiol-modified monomers employed in the all-to-all dimerization networks are reported below:

#### 1.1.1. Thiol-modified DNA monomers (network size = 10) (Fig. 2; S4; S6)

| **Name** | **Sequence 5' → 3'** |
|---|---|
| $M_a$ | ATAGA TCCTG ATAGC - **Thiol C6** |
| $M_b$ | **Thiol C6** - GAGAC CTAAC |
| $M_c$ | GCCAA GTGGA TTGCG TC - **Thiol C6** |
| $M_d$ | **Thiol C6** - TGT TTGTT GAGTA |
| $M_e$ | ACATG CGGAT TG - **Thiol C6** |
| $M_f$ | **Thiol C6** - GTATT GTCTG GT |
| $M_g$ | AGAGG AGGCT - **Thiol C6** |
| $M_h$ | **Thiol C6** - GAGAT TGATC |
| $M_i$ | AGTTG ATGAT GAACC CTT - **Thiol C6** |
| $M_j$ | **Thiol C6** - TTTCA AAGAG ATT |

#### 1.1.2. Input DNA strands (network size = 10) (Fig. 2d; 2g; S4; S6)

| **Name** | **Sequence 5' → 3'** |
|---|---|
| $I_{cd}$ | TACTC AACAA ACAGA CGCAA TCCAC TTGGC |
| $I_{ef}$ | ACCAG ACAAT ACCAA TCCGC ATGT |
| $I_{gh}$ | GATCA ATCTC AGCCT CCTCT |

$I_{ij}$ AATCT CTTTG AAAAA GGGTT CATCA TCAAC T

$I_{ab}$ GTTAG GTCTC GCTAT CAGGA TCTAT

Different combinations of inputs from $I_{cd}$ to $I_{ij}$ were used for experiments with network size = 10 (Fig. 2d). The combinations of input sets used in Figures 2g, S4 and S6 are listed below:

Set #1: $I_{gh}$, $I_{ij}$, $I_{cd}$ and $I_{ef}$.

Set #2: $I_{gh}$, $I_{ij}$, $I_{ef}$ and $I_{ab}$.

Set #3: $I_{gh}$, $I_{ij}$, $I_{cd}$ and $I_{ab}$.

Set #4: $I_{gh}$, $I_{ij}$ and $I_{ef}$.

Set #5: $I_{gh}$, $I_{ij}$ and $I_{cd}$.

Set #6: $I_{gh}$, $I_{ij}$ and $I_{ab}$.

### 1.1.3. Thiol-modified DNA monomers (network size = 4) (Fig. S5)

| **Name** | **Sequence 5' → 3'** |
|---|---|
| $M_a$ | ATAGA TCCTG ATAGC -**Thiol C6** |
| $M_b$ | **Thiol C6** - GAGAC CTAAC |
| $M_c$ | GCCAA GTGGA TTGCG TC - **Thiol C6** |
| $M_d$ | **Thiol C6** -TGT TTGTT GAGTA |

### 1.1.4. Input DNA strand (network size = 4) (Fig. S5)

$I_{cd}$ TACTC AACAA ACAGA CGCAA TCCAC TTGGC

### 1.1.5. Thiol-modified DNA monomers (network size = 30) (Fig. S7)

| **Name** | **Sequence 5' → 3'** |
|---|---|
| $M_a$ | ATAGA TCCTG ATAGC -**Thiol C6** |
| $M_b$ | **Thiol C6** - GAGAC CTAAC |
| $M_c$ | GCCAA GTGGA TTGCG TC - **Thiol C6** |
| $M_d$ | **Thiol C6** - TGT TTGTT GAGTA |
| $M_e$ | ACATG CGGAT TG - **Thiol C6** |
| $M_f$ | **Thiol C6** - GTATT GTCTG GT |
| $M_g$ | AGAGG AGGCT - **Thiol C6** |
| $M_h$ | **Thiol C6** -GAGAT TGATC |
| $M_i$ | AGTTG ATGAT GAACC CTT - **Thiol C6** |
| $M_j$ | **Thiol C6** -TTTCA AAGAG ATT |
| $M_k$ | CAGAC AGTTT CGT - **Thiol C6** |
| $M_l$ | **Thiol C6** - GGTCA TCGTA CCT |
| $M_m$ | GCCAA GTGGA TTGCG TC - **Thiol C6** |
| $M_n$ | **Thiol C6** -TTCTG GAGCG TTGGA CGAAA CT |
| $M_o$ | CTCAG TGGAC AGCCG - **Thiol C6** |
| $M_p$ | **Thiol C6** - GTATT GTCTG GT |
| $M_q$ | AGAGG AGGCT - **Thiol C6** |
| $M_r$ | **Thiol C6** - GAGAT TGATC |
| $M_s$ | AGTTG ATGAT GAACC CTT - **Thiol C6** |
| $M_t$ | **Thiol C6** - TTTCA AAGAG ATT |

| | |
|---|---|
| $M_u$ | ATAGA TCCTG ATAGC - **Thiol C6** |
| $M_v$ | **Thiol C6** - GAGAC CTAAC |
| $M_w$ | GCCAA GTGGA TTGCG TC - **Thiol C6** |
| $M_x$ | **Thiol C6** -TGT TTGTT GAGTA |
| $M_y$ | ACATG CGGAT TG - **Thiol C6** |
| $M_z$ | **Thiol C6** -GTATT GTCTG GT |
| $M_{a'}$ | AGAGG AGGCT - **Thiol C6** |
| $M_{b'}$ | **Thiol C6** - GAGAT TGATC |
| $M_{c'}$ | AGTTG ATGAT GAACC CTT - **Thiol C6** |
| $M_{d'}$ | **Thiol C6** -TTTCA AAGAG ATT |

**1.1.6. Input DNA strands (network size = 30) (Fig. S7)**

| **Name** | **Sequence 5’ → 3’** |
|---|---|
| $I_{ab}$ | GTTAG GTCTC GCTAT CAGGA TCTAT |
| $I_{cd}$ | TACTC AACAA ACAGA CGCAA TCCAC TTGGC |
| $I_{ef}$ | ACCAG ACAAT ACCAA TCCGC ATGT |
| $I_{gh}$ | GATCA ATCTC AGCCT CCTCT |
| $I_{ij}$ | AATCT CTTTG AAAAA GGGTT CATCA TCAAC T |
| $I_{kl}$ | AGGTA CGATG ACCAC GAAAC TGTCT G |
| $I_{mn}$ | AGTTT CGTCC AACGC TCCAG AACGG CTGTC CACTG AG |
| $I_{op}$ | GAAGA TGTCC AGTCG GTCTA CGGAA ATGTC CAATA CG |
| $I_{qr}$ | AAGGA CCAGC TAAAG ACGGA CATT |
| $I_{st}$ | GTAAA TAACA TACAT AATCA AC |
| $I_{uv}$ | ATCGA GTAGG AAATT CTAGA |

| | |
|---|---|
| $I_{wx}$ | GTGGC GTTTT ATTCT ACTAT T |
| $I_{yz}$ | ATCGT CTTAC AGATT CACTT AA |
| $I_{a'b'}$ | TGTTA ATACT GAACT GAGAG A |
| $I_{c'd'}$ | CGAGA GGTGG CTAAC TAATG GTG |

The combinations of input sets used in Figures S7 are listed below:

Set #7: input strands from $I_{gh}$ to $I_{c'd'}$.

Set #6: input strands from $I_{gh}$ to $I_{c'd'}$ + $I_{ab}$.

Set #5: input strands from $I_{gh}$ to $I_{c'd'}$ + $I_{cd}$.

Set #4: input strands from $I_{gh}$ to $I_{c'd'}$ + $I_{ef}$.

Set #3: input strands from $I_{gh}$ to $I_{c'd'}$ + $I_{ab}$+ $I_{cd}$.

Set #2: input strands from $I_{gh}$ to $I_{c'd'}$ + $I_{ab}$ + $I_{ef.}$

Set #1: input strands from $I_{gh}$ to $I_{c'd'}$ + $I_{cd}$ + $I_{ef}$.

For experiments using a network size = 30 a concentration of 3.0 mM of oxidizing agent was employed.

### 1.2. DNA-based dimerization network through SPAAC reaction

The sequences of the SPAAC-modified monomers employed in each many to many dimerization network are reported below:

#### 1.2.1. Azide-modified and DBCO-modified DNA monomers (network size = 20) (Fig. 3; S8)

| **Name** | **Sequence 5' → 3'** |
|---|---|
| $M_a\_N_3$ | ATAGA TCCTG ATAGC **-C3-Azid** |
| $M_b\_N_3$ | **C3-Azid-** GAGAC CTAAC |

| | |
|---|---|
| $M_c\_N_3$ | GCCAA GTGGA TTGCG TC **-C3-Azid** |
| $M_d\_N_3$ | **C3-Azid-** TGTTT GTTGA GTA |
| $M_e\_N_3$ | ACATG CGGAT TG **-C3-Azid** |
| $M_f\_N_3$ | **C3-Azid-** GTATT GTCTG GT |
| $M_g\_N_3$ | AGAGG AGGCT **-C3-Azid** |
| $M_h\_N_3$ | **C3-Azid-** GAGAT TGATC |
| $M_i\_N_3$ | AGTTG ATGAT GAACC CTT **-C3-Azid** |
| $M_j\_N_3$ | **C3-Azid-** TTTCA AAGAG ATT |
| $M_a$_DBCO | ATAGA TCCTG ATAGC **-DBCO** |
| $M_b$_DBCO | **DBCO-** GAGAC CTAAC |
| $M_c$_DBCO | GCCAA GTGGA TTGCG TC **-DBCO** |
| $M_d$_DBCO | **DBCO-** TGTTT GTTGA GTA |
| $M_e$_DBCO | ACATG CGGAT TG **-DBCO** |
| $M_f$_DBCO | **DBCO-** GTATT GTCTG GT |
| $M_g$_DBCO | AGAGG AGGCT **-DBCO** |
| $M_h$_DBCO | **DBCO-** GAGAT TGATC |
| $M_i$_DBCO | AGTTG ATGAT GAACC CTT **-DBCO** |
| $M_j$_DBCO | **DBCO-** TTTCA AAGAG ATT |

### 1.2.2. Input DNA strands (network size = 20) (Fig. 3d; 3f and S8)

| **Name** | **Sequence 5' → 3'** |
|---|---|
| $I_{cd}$ | TACTC AACAA ACAGA CGCAA TCCAC TTGGC |
| $I_{ef}$ | ACCAG ACAAT ACCAA TCCGC ATGT |
| $I_{gh}$ | GATCA ATCTC AGCCT CCTCT |
| $I_{ij}$ | AATCT CTTTG AAAAA GGGTT CATCA TCAAC T |
| $I_{ab}$ | GTTAG GTCTC GCTAT CAGGA TCTAT |

Different combinations of inputs from $I_{cd}$ to $I_{ji}$ were used for experiments in Fig. 3d. The combinations of input sets used in Figures 3f and S6 are listed below:

Set #1: $I_{gh}$, $I_{ij}$, $I_{cd}$ and $I_{ef}$.

Set #2: $I_{gh}$, $I_{ij}$, $I_{ef}$ and $I_{ab}$.

Set #3. $I_{gh}$, $I_{ij}$, $I_{cd}$ and $I_{ab}$.

Set #4: $I_{gh}$, $I_{ij}$ and $I_{ef}$.

Set #5: $I_{gh}$, $I_{ij}$ and $I_{cd}$.

Set #6: $I_{gh}$, $I_{ij}$ and $I_{ab}$.

Set #7: $I_{gh}$ and $I_{ij}$.

### 1.3. Dimer Output Reporters

To quantify the yield of the dimer outputs we employed 3 orthogonal DNA duplex-reporters. Each reporter is designed to detect a specific dimer output through a strand displacement reaction, resulting in the increase of the observed fluorescence signal (Fig. S2a). The following oligonucleotides were used to form the duplex reporters:

| **Name** | **Sequence 5' → 3'** |
|---|---|
| Rep#1_ $D_{ab}$_cy3 | **Cy3-** ATAGA TCCTG ATAGC GAGAC |
| Rep#1_ $D_{ab}$_BHQ-2 | GTTAG GTCTC GCTAT CAGGA TCTAT **- BHQ-2** |
| Rep#2_ $D_{cd}$_cy5 | **Cy5-** GCCAA GTGGA TTGCG TCTGT TTG |
| Rep#2_ $D_{cd}$_BHQ-2 | TACTC AA CAAAC AGACG CAATC CACTT GGC**-BHQ-2** |
| Rep#3_ $D_{ef}$_FAM | **6-FAM-** ACATG CGGAT TGGTA TTGT |
| Rep#3_ $D_{ef}$_BHQ-1 | ACCAG ACAAT ACCAA TCCGC ATGT**-BHQ-1** |

**Target Dimer outputs**

| | |
|---|---|
| $D_{ab}$ | ATAGA TCCTG ATAGC **-Thiol C6 SS -** GAGAC CTAAC |

| | |
|---|---|
| $D_{cd}$ | GCCAA GTGGA TTGCG TC -**Thiol C6 SS -** TGT TTGTT GAGTA |
| $D_{ef}$ | ACATG CGGAT TG -**Thiol C6 SS -** GTATT GTCTG GT |

Reporter #1 is specific for dimer output $D_{ab}$, Reporter #2 for $D_{cd}$, and Reporter #3 for $D_{ef}$. The underlined bases indicate the toehold domain.

### 1.4. DNA-based structures experiments (Fig. 4)

#### 1.4.1. DNA tile-forming strands:

The following oligonucleotides were used as tile-forming strands for the three different DNA-based structures employed in this work:

**DNA structure #1**

| **Name** | **Sequence 5' → 3'** |
|---|---|
| S1_#1 | ATAGA TCCTG ATAGC GAGAC CTAGC AACCT GAAAC CA |
| S2_#1 | <u>TCTCA GC</u> **CTCCT** CTACT CGTGG ATCTA T **AAGGT** |
| S3Cy3_#1 | **Cy3-** AGAAT TGCGT CGTGG TTGCT AGGTC TCGCT ATCAC CGATG TG |
| S3Cy5_#1 | **Cy5-** AGAAT TGCGT CGTGG TTGCT AGGTC TCGCT ATCAC CGATG TG |
| S4_#1 | **AGGAG** TGGTT TCACC TTAAC G **ACCTT** |
| S5_#1 | CGTTA AGGAC GACGC AATTC TCACA TCGGA CGAGT AG |

**DNA structure #2**

| **Name** | **Sequence** |
|---|---|
| S1_#2 | CT AG TGGAC AGCCG TTCTG GAGCG TTGGA CGAAA CT |
| S2_#2 | <u>TGGTA TT</u> **GTCTG** GTAGA GCACC ACTGA G **AGGTA** |

| | |
|---|---|
| S3_#2 | **FAM-** TCCAG AACGG CTGTG GCTAA ACAGT AACCG AAGCA CCAAC GCT |
| S4_#2 | **CAGAC** AGTTT CGTGG TCATC G **TACCT** |
| S5_#2 | CGATG ACCTG CTTCG GTTAC TGTTT AGCCT GCTCT AC |

**DNA structure #3**

| Name | Sequence |
|---|---|
| S1_#3 | CGTAT TGGAC ATTTC CGTAG ACCGA CTGGA CATCT TC |
| S2_#3 | <u>TCTTT AG</u> **CTGGT** CCTTC ACACC AATAC G **GCATT** |
| S3_#3 | **Cy3-** TTCTA CGGAA ATGTG GCAGA ATCAA TCATA AGACA CCAGT CGG |
| S4_#3 | **ACCAG** GAAGA TGTGG TAGTG G **AATGC** |
| S5_#3 | CCACT ACCTG TCTTA TGATT GATTC TGCCT GTGAA GG |

The sequences above represent the DNA strands (S1-S5) that form the three orthogonal DNA tiles. In strands S2 and S4, nucleotides in bold indicate the sticky end portions, while those underlined in strand S2 represent the regulatory binding domain. Each of the three orthogonal DNA tiles features unique sticky ends and a distinct regulatory binding domain. Additionally, each strand S3 is conjugated at the 5' end with orthogonal fluorophores: Cyanine-3 (Cy3) or Cyanine-5 (Cy5) for system #1, Cyanine-3 (Cy3) for system #2, and Fluorescein (6-FAM) for system #3 (Fig. S9; S10).

### 1.4.2. DNA monomers to control the disassembly of DNA structure #1 (network size = 10)(Fig. 4b)

| Name | Sequence 5' → 3' |
|---|---|
| $M_{\_\#1\_1}$ | AGAGG AG - **Thiol C6** |

| | |
|---|---|
| $M_{\_\#1\_2}$ | **Thiol C6** - GCTGA GA |
| $M_a$ | ATAGA TCCTG ATAGC - **Thiol C6** |
| $M_b$ | **Thiol C6** - GAGAC CTAAC |
| $M_c$ | GCCAA GTGGA TTGCG TC - **Thiol C6** |
| $M_d$ | **Thiol C6** - TGT TTGTT GAGTA |
| $M_e$ | ACATG CGGAT TG - **Thiol C6** |
| $M_f$ | **Thiol C6** - GTATT GTCTG GT |
| $M_g$ | CAGAC AGTTT CGT - **Thiol C6** |
| $M_h$ | **Thiol C6** - GGTCA TCGTA CCT |

Monomers, $M_{\#1\_1}$ and $M_{\#1\_2}$ dimerize to form the invader output, $D_{\#1}$. $D_{\#1}$ can bind the regulatory binding domain of the DNA tiles, invading one of the sticky ends resulting in the DNA structure #1 disassembly (Fig. S9c). [1,2]

### 1.4.3. Input DNA strands (Fig. 4b)

In Fig. 4b, the input set comprises all of the sequences listed below.

| **Name** | **Sequence 5' → 3'** |
|---|---|
| $I_{ab}$ | GTTAG GTCTC GCTAT CAGGA TCTAT |
| $I_{cd}$ | TACTC AACAA ACAGA CGCAA TCCAC TTGGC |
| $I_{ef}$ | ACCAG ACAAT ACCAA TCCGC ATGT |
| $I_{gh}$ | AGGTA CGATG ACCAC GAAAC TGTCT G |

### 1.4.4. DNA dimerization networks monomers for the orthogonal control of the disassembly of DNA structures #1, #2 and #3 (network size = 10)(Fig. 4d)

| **Name** | **Sequence 5' → 3'** |
|---|---|

| | |
|---|---|
| $M_{\_\#1\_1}$ | AGAGG AG - **Thiol C6** |
| $M_{\_\#1\_2}$ | **Thiol C6** - GCTGA GA |
| $M_{\_\#2\_1}$ | CTACC AGAC - **Thiol C6** |
| $M_{\_\#2\_2}$ | **Thiol C6** - AATAC CAAA |
| $M_{\_\#3\_1}$ | AAGGA CCAG - **Thiol C6** |
| $M_{\_\#3\_2}$ | **Thiol C6** - CTAAA GA |
| $M_a$ | ATAGA TCCTG ATAGC -**Thiol C6** |
| $M_b$ | **Thiol C6** - GAGAC CTAAC |
| $M_c$ | GCCAA GTGGA TTGCG TC - **Thiol C6** |
| $M_d$ | **Thiol C6** - TGT TTGTT GAGTA |

Dimer formed by interaction of $M_{\#1\_1}$ and $M_{\#1\_2}$ disassemble structure #1. Dimers formed by $M_{\#2\_1}$ + $M_{\#2\_2}$ and $M_{\#3\_1}$ + $M_{\#3\_2}$ act as invader outputs for the disassembly of DNA structures #2 and #3, respectively.

### 1.4.5. Input DNA strands (Fig. 4d)

The sequences used as inputs for thiol-based DNA networks in Fig. 4d are reported below:

| **Name** | **Sequence 5' → 3'** |
|---|---|
| $I_{\#1}$ | TCTCA GCCTC CTCT |
| $I_{\#2}$ | TTTGG TATTG TCTGG TAG |
| $I_{\#3}$ | TCTTT AGCTG GTCCT T |
| $I_{ab}$ | GTTAG GTCTC GCTAT CAGGA TCTAT |
| $I_{cd}$ | TACTC AACAA ACAGA CGCAA TCCAC TTGGC |

Set #1 is formed by $I_{ab}$, $I_{cd}$, $I_{\#2}$ and $I_{\#3}$

Set #2 is formed by $I_{ab}$, $I_{cd}$, $I_{\#1}$ and $I_{\#3}$.

Set #3 is formed by $I_{ab}$, $I_{cd}$, $I_{\#1}$ and $I_{\#2}$.

### 1.4.6. Invader Dimer control strands

The following control dimer strands were used as invaded dimer control strands:

| | |
|---|---|
| $D_{\#1}$ | AGAGG AG -**Thiol C6 SS -** GCTGA GA |
| $D_{\#2}$ | CTACC AGAC -**Thiol C6 SS -** AATAC CAAA |
| $D_{\#3}$ | AAGGA CCAG -**Thiol C6 SS -** CTAAA GA |

## 2. Fluorescence Experiments

Fluorescence experiments were carried out on a Tecan F200pro plate reader using the top reading mode with black, flat bottom non-binding 384-well plates and a 30 µL final volume of the dimerization network solution. The working wavelengths were set to $\lambda_{ex}$ = 548 (± 9) nm and $\lambda_{em}$ = 589 (± 20) nm for the Cy3 labeled oligonucleotides (Reporter #1, $\lambda_{ex}$ = 646 (± 9) nm and $\lambda_{em}$ = 685 (± 20) nm for the Cy5 labeled oligonucleotide (Reporter #2) and $\lambda_{ex}$ = 490 (± 9) nm and $\lambda_{em}$ = 525 (± 20) nm for the FAM labeled oligonucleotide (Reporter #3).

## 3. Data Analysis

Quantitative analysis was performed using GraphPad Prism 8 software. No specific preprocessing of data was performed prior to statistical analyses.

## 4. Quantification of dimer output ($D_{ij}$) concentration

To quantify the concentration of the target dimer output, we have performed calibration curves by spiking different standard concentrations of the target dimer outputs (e.g. $D_{ab}$,

$D_{cd}$, $D_{ef}$) in a solution mixture containing all the monomers of the DNA-network (Fig. S2b). The resulting fluorescence signals were fitted with the following Hill equation:

$$F(T) = F_0 + \left( \frac{[T]^h (F_B - F_0)}{[T]^h + K_{1/2}^h} \right)$$

Where F(T) is the fluorescence in presence of different concentration of target dimer output, $F_0$ is the background fluorescence, [T] is the concentration of the target dimer output, $F_B$ is the fluorescence in the presence of saturating concentration of target, and *h* is the Hill coefficient.

We note, however, that the above method does not allow to reliably quantify dimer output concentrations that fall outside the linear dynamic range of the calibration curve. In such cases, to quantify the unknown concentration of the target dimer output formed in the DNA-based dimerization network, we have employed a method that adapts the standard addition method to assays with non-linear calibration curves.[3]

To do this, we linearized the calibration curves described above by plotting the logit values against the logarithm of the estimated total concentration of target dimer output, where the logit function is defined as follows:

$$logit\ (y) = ln\ \frac{y}{1 - y}$$

Where $y$ represents the normalized relative fluorescence signal and the estimated total concentration of target is defined as the sum between the spiked standard concentration and guess concentrations between 0 and 200 nM. For each guessed value we then calculated the linear regression of the logit function vs the logarithm of total concentration (standard concentration + guess) and evaluated its deviation from linearity using the residual sum of squares ($SS_{res}$). The guess value corresponding to the minimum value of $SS_{res}$ represents the unknown concentration of the target dimer output formed in the mixture (Fig. S2c-d).

## 5. Quantification of the "Relative Assembled tile density"

To quantify the assembled tile density, we have analyzed the fluorescence microscopy images and calculated the number of the formed polymers (count) and their mean length (<L>, nm) using SPIP Software - Scanning Probe Image Processor. These two values were then employed to calculate the "length occupied by all active tiles":

$$Length\ of\ all\ active\ tiles, nm\ = count \cdot\ < Length, nm >$$

Then, by considering a length of 14.3 nm for a single DNA tile[4] we calculated the number of the activated tiles over the length:

$$Active\ tiles\ over\ length = \frac{Length\ of\ activated\ tiles, nm}{14.3\ nm}$$

Due to the tubular structure of our DNA structure, we can consider an average of 7 DNA tiles per circumference,[4] so we can obtain the total number of assembled tiles by multiplying the activated tiles over length by 7:

$$Assembled\ tiles = Active\ tiles\ over\ length \cdot\ 7$$

Finally, to calculate the assembled tiles density we divided the total assembled tiles calculated above by the value of the examined area (0.01532 $mm^2$):

$$Assembled\ tiles\ density = \frac{Assembled\ tiles}{0.01532\ \ mm^2}$$

For all these values, the measurements have been performed in triplicate independent experiments and the error bars reflect the standard deviations.

Starting from this definition we were able to determine the "Relative assembled tile density", that is defined as the ratio between the assembled tile density in the sample and the assembled tile density in absence of inputs.

## 6. Supplementary Figures

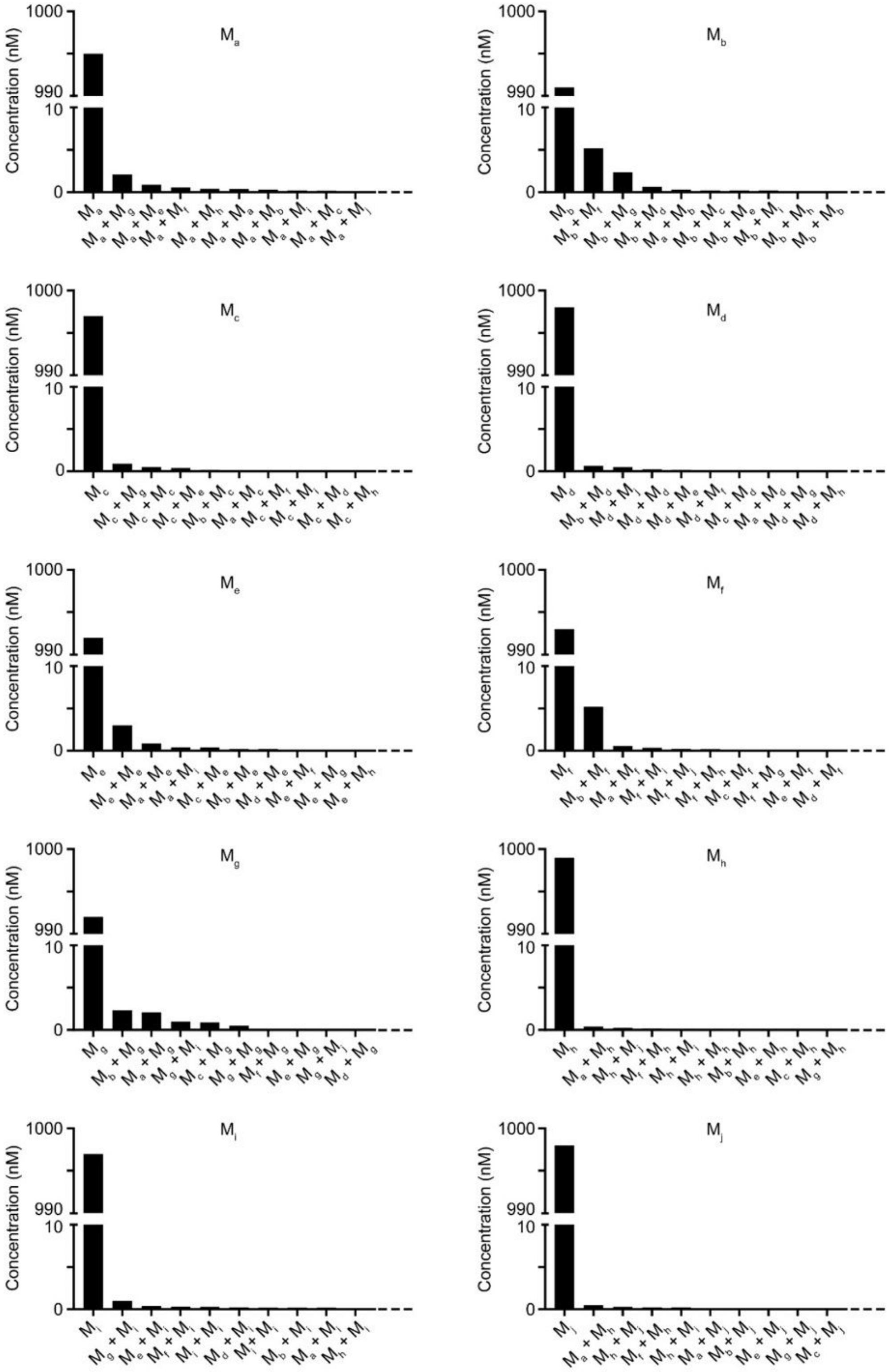

**Figure S1.** Predicted concentration values of each monomer ($M_i$) and of non-specific duplexes ($M_i$+$M_j$) that can form in a mixture containing the 10 DNA-based monomers used in this work (Figures 2 and 3). The concentration values were calculated using NUPACK software. Each monomer was set at a total initial concentration of 1 μM.

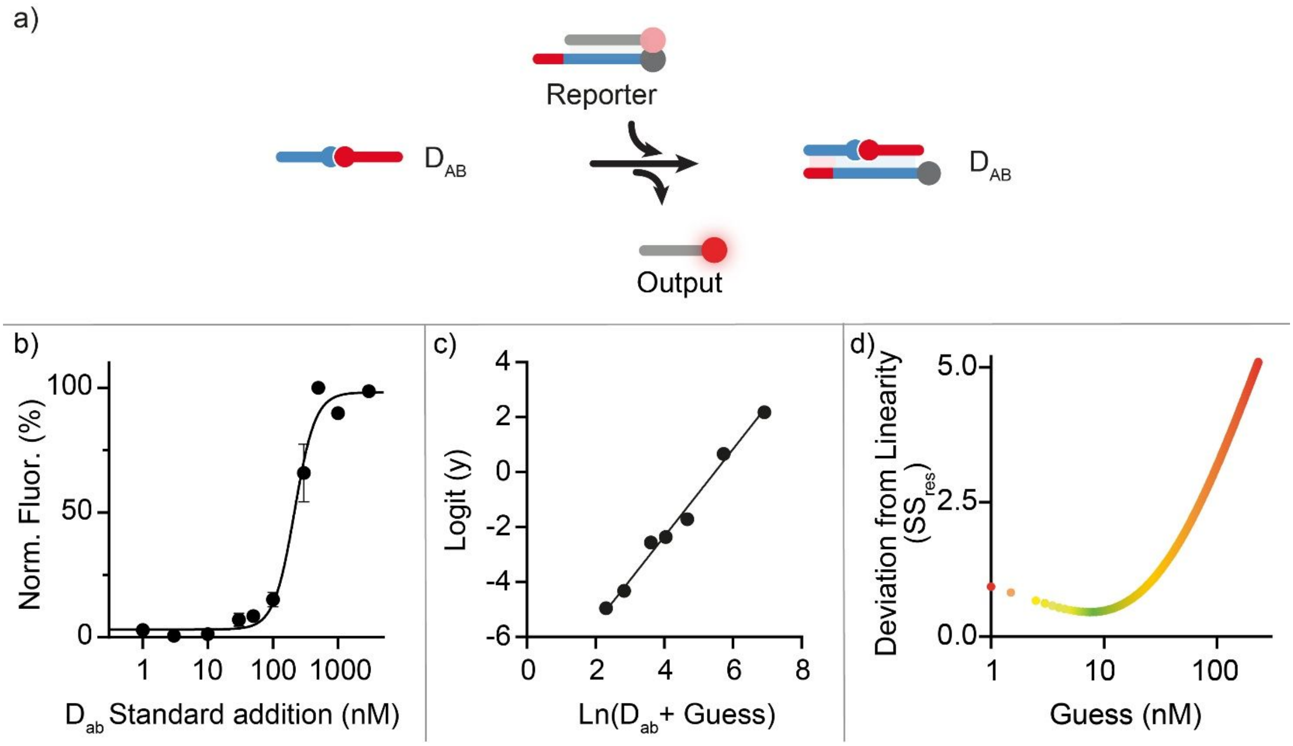


**Figure S2.** a) Scheme of the strand displacement reaction (SD) used for the detection of target dimer outputs. b) Example of the calibration curve obtained for reporter #1 for the quantification of target dimer output $D_{ab}$. This curve was generated by adding the reporter (0.50 μM) and different standard concentrations of the target dimer output in the reaction mixture (here network size = 10) after the completion of the network's reaction (72 hours). The signal was measured after 6 hours from the addition of the reporter and the standard target dimer output. c) Example of linearization of the calibration curve by plotting the logit values against the logarithm of the estimated total concentration of target dimer output (here only one guessed concentration value of dimer output is shown). d) Plot of the residual sum of squares ($SS_{res}$) used as an indicator of the deviation from linearity for each guess concentration (nM) vs the guess concentration. The concentration value corresponding to the minimum value of $SS_{res}$ represents the unknown concentration of the target dimer output. The experiments shown in this figure were performed in triplicate in 1×TAE, 12.5 mM $MgCl_2$, pH 8.5. Error bars represent the standard deviation based on triplicate measurements.

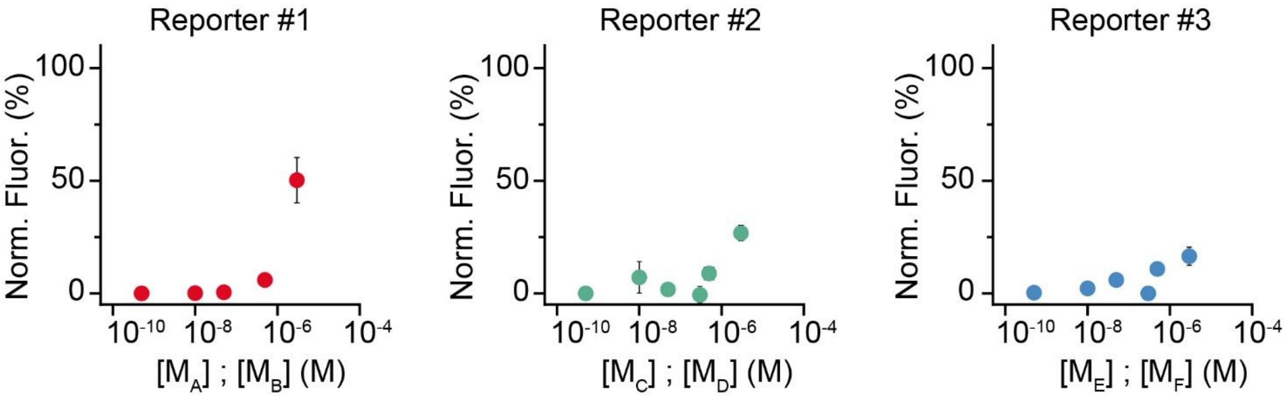


**Figure S3.** Fluorescence signals vs thiol-DNA monomer concentrations. The results demonstrate that each reporter is not activated by single monomers. These experiments were performed using a concentration of 0.50 µM of reporter and adding increasing concentration of the indicated pair of monomers. The experiments shown in this figure were performed in triplicate in 1×TAE, 12.5 mM $MgCl_2$, pH 8.5. Error bars represent the standard deviation based on triplicate measurements.

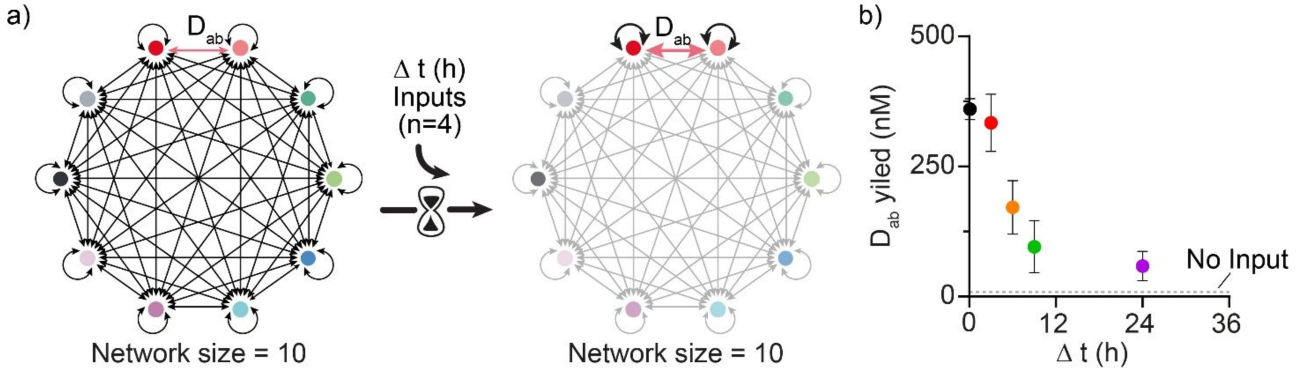


**Figure S4.** a) Scheme of a thiol-DNA based dimerization network consisting of 10 monomers where the inputs (n = 4) are introduced at different times after the initiation of the dimerization process. b) Modulation of the relative yield of $D_{ab}$ output at different input addition time (0, 3, 6, 9, 24 h) after the initiation of the dimerization process. The experiments shown in this figure were performed in triplicate in 1×TAE, 12.5 mM $MgCl_2$, pH 8.5. Each monomer is at a concentration of 1.0 µM. Inputs are added at equimolar concentration (1.0 µM). Error bars represent the standard deviation based on triplicate measurements.

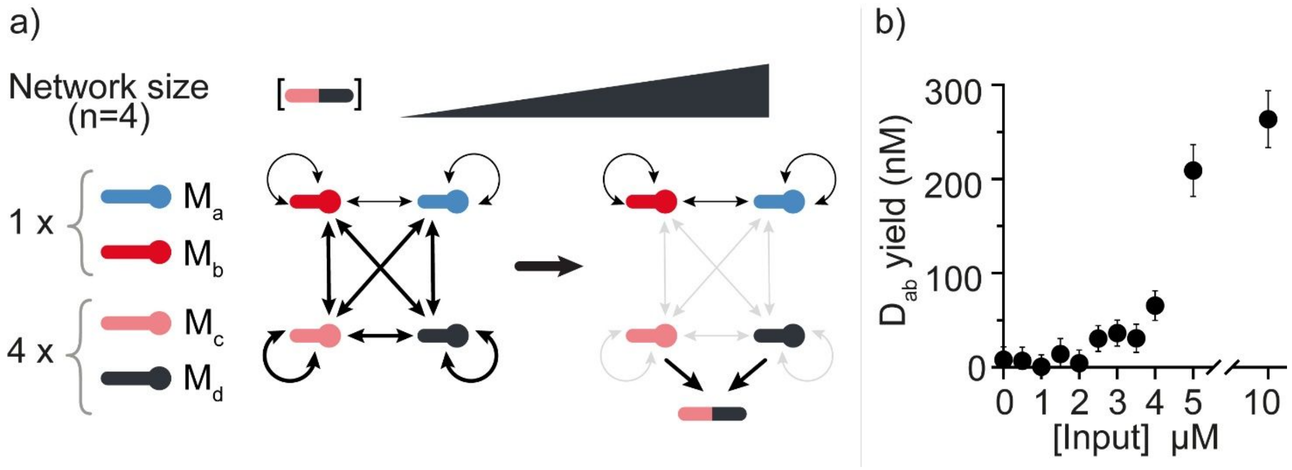


**Figure S5.** a) Scheme of a thiol-DNA based dimerization network consisting of 4 monomers: $M_a$, $M_b$, $M_c$ and $M_d$. $M_a$ and $M_b$ are present at a concentration of 1.0 μM, while $M_c$ and $M_d$ at a concentration of 4.0 μM. the input DNA strand excludes $M_c$ and $M_d$ monomers. b) $D_{ab}$ yield in nM at increasing concentration of input strand. The experiments shown in this figure were performed in triplicate in 1×TAE, 12.5 mM $MgCl_2$, pH 8.5. Error bars represent the standard deviation based on triplicate measurements.

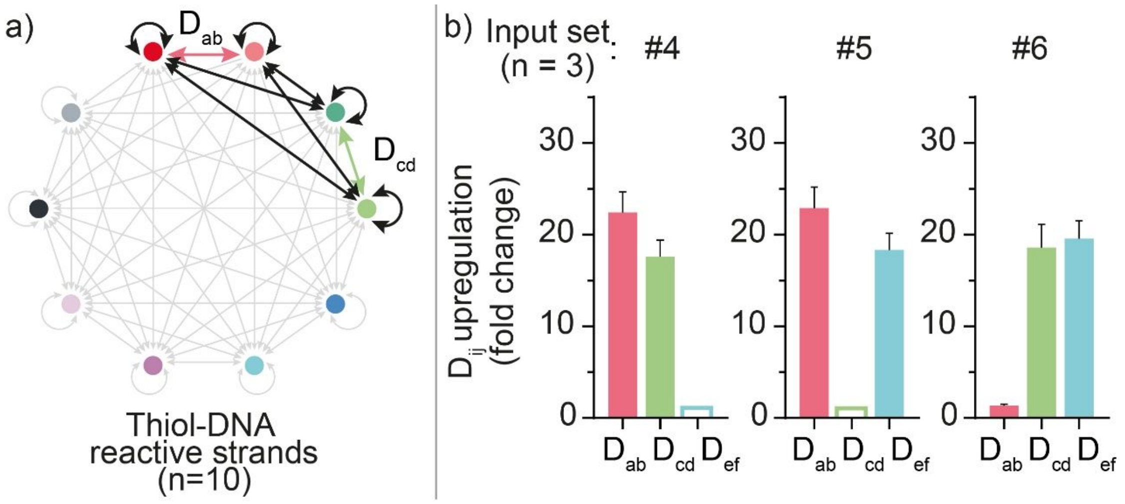


**Figure S6.** a) Scheme of a thiol-DNA based dimerization network consisting of 10 monomers when 3 inputs are present. b) Dimer output upregulation using different sets of inputs. The experiments shown in this figure were performed in 1×TAE, 12.5 mM $MgCl_2$, pH 8.5. Each monomer is at a concentration of 1.0 μM. Inputs are added at equimolar concentration (1.0 μM). Error bars represent the standard deviation based on triplicate measurements.

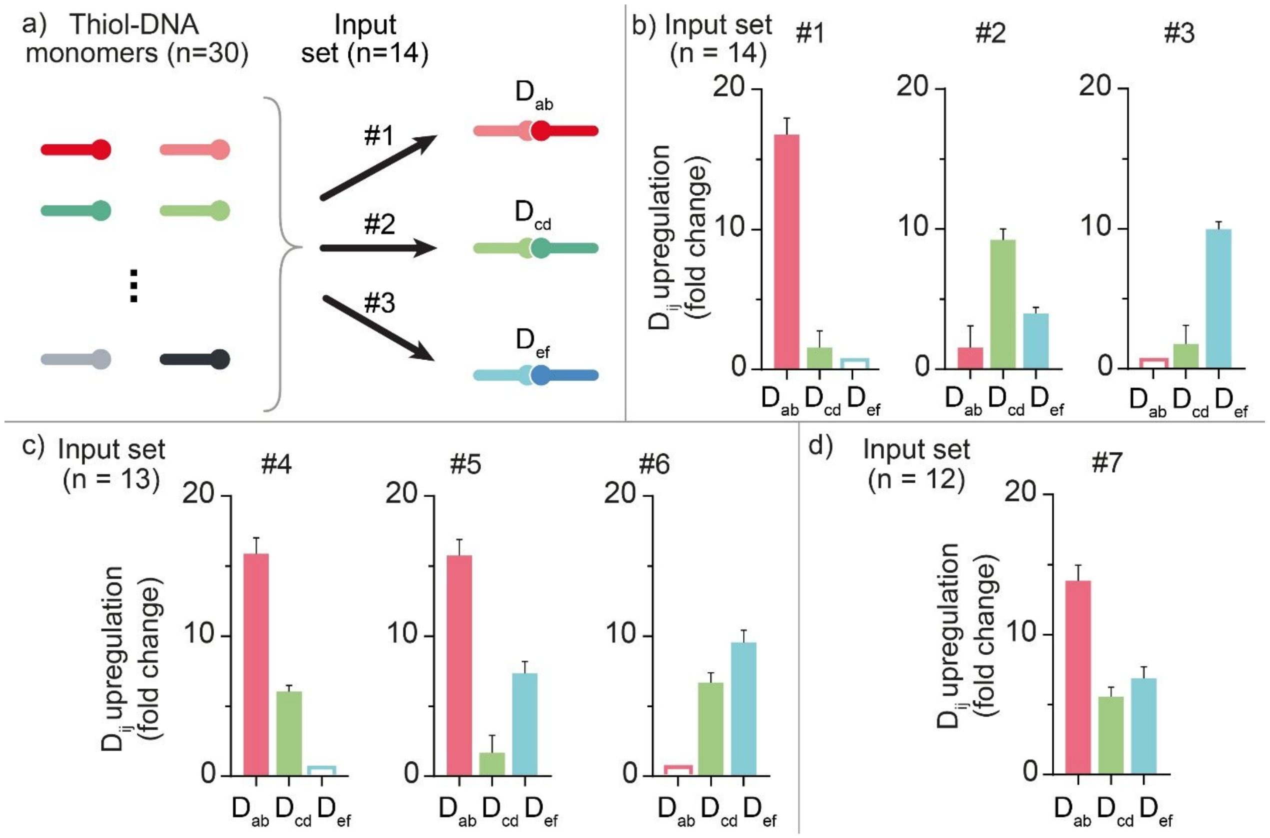


**Figure S7.** a) Scheme of a thiol-DNA based dimerization network consisting of 30 monomers. b-d) Dimer output upregulation using different sets of inputs. The experiments shown in this figure were performed in triplicate in 1×TAE, 12.5 mM $MgCl_2$, pH 8.5. Each monomer is at a concentration of 1.0 μM. Inputs are added at equimolar concentration (1.0 μM). Error bars represent the standard deviation based on triplicate measurements.

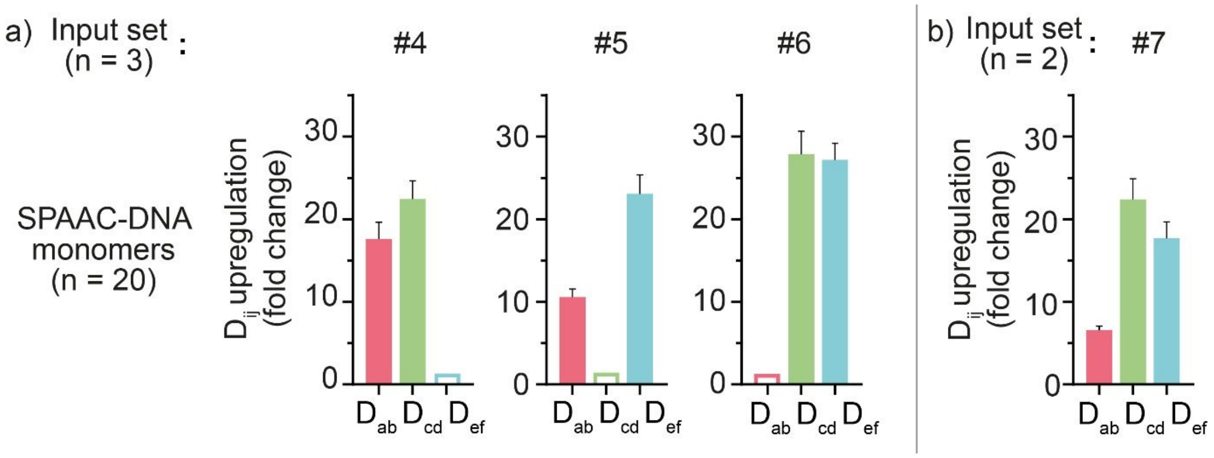


**Figure S8.** a-b) Dimer output upregulation using different combinations of inputs with the dimerization network shown in Figure 3 (network size = 20). In panel a the inputs are designed to upregulate 2 dimer outputs. In panel b the inputs are designed to upregulate 3 dimer outputs. The experiments shown in this figure were performed in carbonate buffer 50 mM $NaHCO_3$, 1.0 M NaCl, pH 8.6. Each monomer is at a concentration of 0.50 μM. Inputs are added at a concentration of 1.0 μM. Error bars represent the standard deviation based on triplicate measurements.

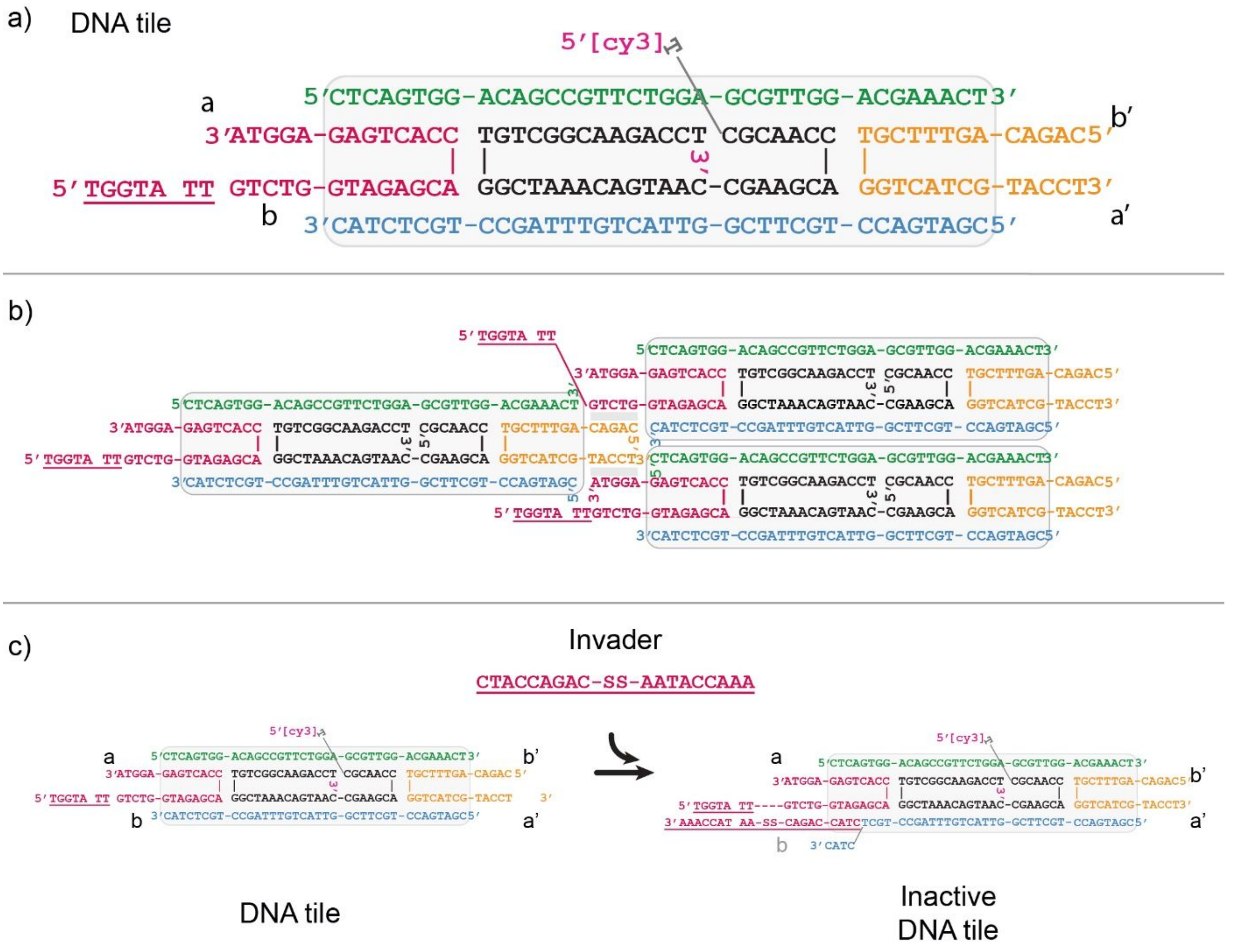


**Figure S9.** a) Scheme of the five strands forming the tile with fluorophore. The fluorophore (in pink) is conjugated to the 5'-end of the central strand, S3. The underlined nucleotides denote the regulatory binding domain. b) Scheme showing how DNA tiles hybridize to form the DNA-based structure. The sticky ends are denoted as a, a', b and b'. c) Scheme showing DNA tile inactivation in presence of invader dimer output.[1,2]

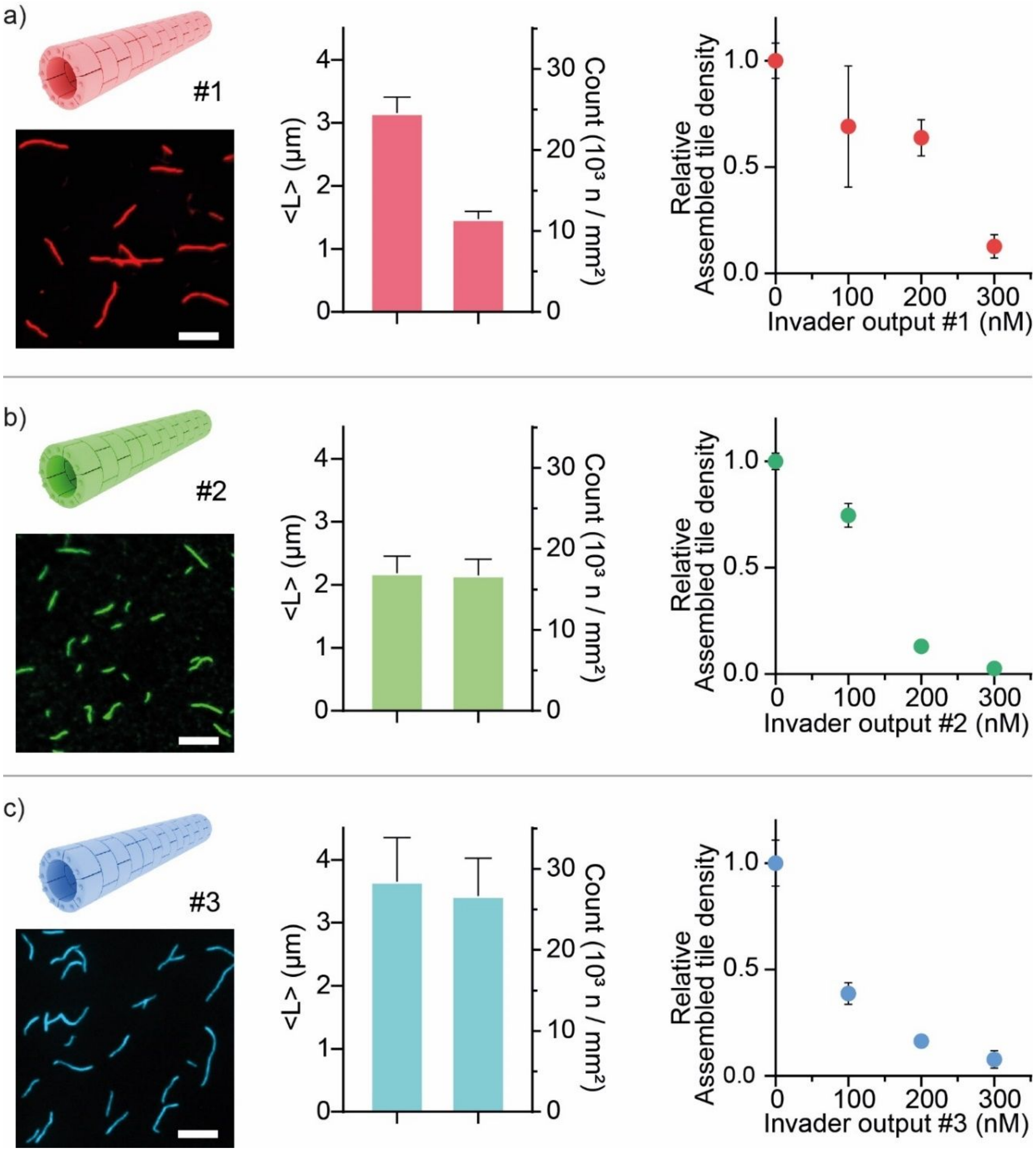


**Figure S10.** a-c) left) Fluorescence images of the three DNA structures. Middle) Bar plots showing average length (μm) and count (count $10^3$ count/ $mm^2$) of each DNA structure. Right) Relative assembled tile density in response to invader output concentration ($D_{\#1}$, $D_{\#2}$ and $D_{\#3}$). The experiments shown in this figure were performed in triplicate in 1×TAE, 12.5 mM $MgCl_2$, pH 8.5. For disassembly experiments a concentration of 100 nM of DNA structure was used. Error bars represent the standard deviation based on triplicate measurements. Scale bars: 5 μm.

## 7. References


(1) Green, L. N.; Subramanian, H. K. K.; Mardanlou, V.; Kim, J.; Hariadi, R. F.; Franco, E. Autonomous Dynamic Control of DNA Nanostructure Self-Assembly. *Nat. Chem.* **2019**, *11* (6), 510–520. https://doi.org/10.1038/s41557-019-0251-8.

(2) Gentile, S.; Del Grosso, E.; Prins, L. J.; Ricci, F. Reorganization of Self-Assembled DNA-Based Polymers Using Orthogonally Addressable Building Blocks. *Angew. Chem., Int. Ed.* **2021**, *60* (23), 12911–12917. https://doi.org/10.1002/anie.202101378.

(3) Conrad, M.; Fechner P.; Proll G.; Gauglitz G. (R)evolution of the Standard Addition Procedure for Immunoassays. *Biosensors*. **2023**, *13*, (9), 849. doi: 10.3390/bios13090849.

(4) Rothemund, P. W. K.; Ekani-Nkodo, A.; Papadakis, N.; Kumar, A.; Fygenson, D. K.; Winfree, E. Design and Characterization of Programmable DNA Nanotubes. *J. Am. Chem. Soc.* **2004**, *126* (50), 16344–16352. https://doi.org/10.1021/ja044319l.
